# Autophagic dysfunction and gut microbiota dysbiosis cause chronic immune activation in a *Drosophila* model of Gaucher disease

**DOI:** 10.1371/journal.pgen.1011063

**Published:** 2023-12-21

**Authors:** Magda L. Atilano, Alexander Hull, Catalina-Andreea Romila, Mirjam L. Adams, Jacob Wildfire, Enric Ureña, Miranda Dyson, Jorge Ivan-Castillo-Quan, Linda Partridge, Kerri J. Kinghorn

**Affiliations:** 1 UCL Institute of Healthy Ageing, Department of Genetics, Evolution and Environment, University College London, London, United Kingdom; 2 Section on Islet Cell & Regenerative Biology, Joslin Diabetes Center and Department of Genetics, Harvard Medical School, Boston, United States of America; The University of North Carolina at Chapel Hill, UNITED STATES

## Abstract

Mutations in the *GBA1* gene cause the lysosomal storage disorder Gaucher disease (GD) and are the greatest known genetic risk factors for Parkinson’s disease (PD). Communication between the gut and brain and immune dysregulation are increasingly being implicated in neurodegenerative disorders such as PD. Here, we show that flies lacking the *Gba1b* gene, the main fly orthologue of *GBA1*, display widespread NF*-k*B signalling activation, including gut inflammation, and brain glial activation. We also demonstrate intestinal autophagic defects, gut dysfunction, and microbiome dysbiosis. Remarkably, modulating the microbiome of *Gba1b* knockout flies, by raising them under germ-free conditions, partially ameliorates lifespan, locomotor and immune phenotypes. Moreover, we show that modulation of the immune deficiency (IMD) pathway is detrimental to the survival of *Gba1* deficient flies. We also reveal that direct stimulation of autophagy by rapamycin treatment achieves similar benefits to germ-free conditions independent of gut bacterial load. Consistent with this, we show that pharmacologically blocking autophagosomal-lysosomal fusion, mimicking the autophagy defects of *Gba1* depleted cells, is sufficient to stimulate intestinal immune activation. Overall, our data elucidate a mechanism whereby an altered microbiome, coupled with defects in autophagy, drive chronic activation of NF*-k*B signaling in *a Gba1* loss-of-function model. It also highlights that elimination of the microbiota or stimulation of autophagy to remove immune mediators, rather than prolonged immunosuppression, may represent effective therapeutic avenues for *GBA1-*associated disorders.

## Author summary

Alterations in gut microbes are increasingly being linked to neurodegenerative disorders such as Parkinson’s disease (PD). Mutations in the *GBA1* gene cause Gaucher disease (GD), and are the greatest genetic risk factor for PD. Here we show that *Drosophila* models lacking the fly orthologue of *GBA1* (*Gba1b*) have an altered gut microbiome, which triggers a widespread innate immune response. Accordingly, we show that removal of gut bacteria using antibiotics leads to a reduction in the immune response and health benefits. Stimulating innate immunity, specifically the NF-*k*B/IMD pathway, shortens the survival of *Gba1b* deficient flies, suggesting that the overactive immune system is toxic. Conversely, genetic removal of this branch of the immune response is harmful and associated with increased gut bacteria. Lastly, we identify widespread defects in a process called autophagy, which helps degrade and recycle cellular waste. Pharmacologically boosting autophagy extends survival and lowers the gut immune response without changing the gut bacteria. Taken together, our findings suggest that the gut microbiome and autophagy defects in *Gba1b* deficient flies trigger an abnormal immune response. Thus, drugs which alter the gut microbiome or stimulate autophagy may offer potential therapeutic benefits to patients with *GBA1-*associated disorders.

## Introduction

The *GBA1* gene encodes the lysosomal enzyme glucocerebrosidase (GCase), responsible for the hydrolysis of the key membrane sphingolipid glucosylceramide (GluCer) to produce glucose and ceramide. Bi-allelic mutations in the *GBA1* gene are known to cause Gaucher disease (GD), whereas heterozygous mutations represent a major genetic risk factor for Parkinson’s disease (PD), conferring an approximately 20-fold increased risk [1, 2]. GD is a multi-systemic disorder characterised by the lysosomal accumulation of GluCer within macrophages of the liver, spleen, bone marrow, and other tissues, causing widespread organ dysfunction and inflammation [3]. PD is characterised neuropathologically by the presence of intraneuronal inclusions called Lewy bodies, composed predominantly of aggregated α-synuclein (αSyn), which forms higher-order aggregates in the presence of GluCer, leading to further lysosomal dysfunction [4]. Another pathological hallmark of PD is dopaminergic neuronal loss in the substantia nigra, where widespread GCase deficiency is also observed [5–7].

There is growing evidence to suggest that the gut-brain axis is involved in the development of PD and other neurodegenerative disorders, and that the gut may act as an initiating site for PD pathology [8, 9]. Moreover, studies spanning clinical and basic science research have demonstrated both central and peripheral immune changes in PD and GD, implicating the immune system in gut-brain axis communication. Multiple genetic studies have linked PD risk to immune-related pathways and pathogenic variants in immune genes (e.g., Toll-like receptor (TLR-4), Tumour Necrosis Factor (TNF)-α and Human Leukocyte Antigen-DR (HLA-DR) [10–13]. Furthermore, impaired autophagy in macrophages derived from peripheral monocytes of GD patients leads to increased secretion of the pro-inflammatory cytokines interleukin (IL)-6 and IL-β in association with inflammasome activation [14]. Despite the growing evidence linking immune dysfunction to sporadic PD and GD, the precise role of immune responses in gut-brain cross-talk in *GBA1*-PD and neuronopathic GD is yet to be elucidated.

In *Drosophila*, the innate immune response is initiated by pattern recognition receptors (PRRs), which recognise pathogen associated molecular patterns (PAMPs) on the surface of microbes to induce the conserved immune signal transduction pathways: Immune deficiency (IMD), Toll and JAK/STAT [15, 16]. Activation of innate immune pathways results in the up regulation of a cadre of structurally diverse antimicrobial peptides (AMPs) with bactericidal properties. Nevertheless, chronic immune pathway activation is deleterious to health and thus activation of the innate immune pathways must be tightly regulated [17]. Autophagy is a biological process responsible for the removal of damaged organelles, aggregated proteins, lipids and PAMPs. During autophagy, the cellular material to be degraded is sequestered into double-membraned vesicles called autophagosomes. At a later stage, these structures fuse with lysosomes containing hydrolases, which degrade the sequestered cargo [18].

Here, we describe widespread innate immune activation and gut dysfunction in a *Gba1b* knockout fly model (Kinghorn et al., 2016). Remarkably, we report that the intestinal microbiota is necessary for immune activation, and that eradication of the altered gut microbiome in *Gba1b* knockout flies improves lifespan, locomotor and inflammatory phenotypes. Moreover, we demonstrate that direct stimulation of autophagy with rapamycin is sufficient to reduce immune activation and to partially rescue the GCase-deficient lifespan phenotype. Lastly, we show that modulation of the NF*-k*B/IMD pathway, through overexpression and knockout of *Relish* (*Rel*), is detrimental to flies lacking *Gba1b*, suggesting that chronic manipulation of these pathways should be approached with caution in *GBA1*-related disease. Together, these results suggest that gut microbes play an important role in triggering the immune response in *GBA1*-associated disorders, and that targeting the gut microbiome or improving the autophagic clearance of PAMPs may represent a potential therapeutic strategy in these disorders.

## Results

### Loss of *Gba1b* results in innate immune activation

**Fig 1:**
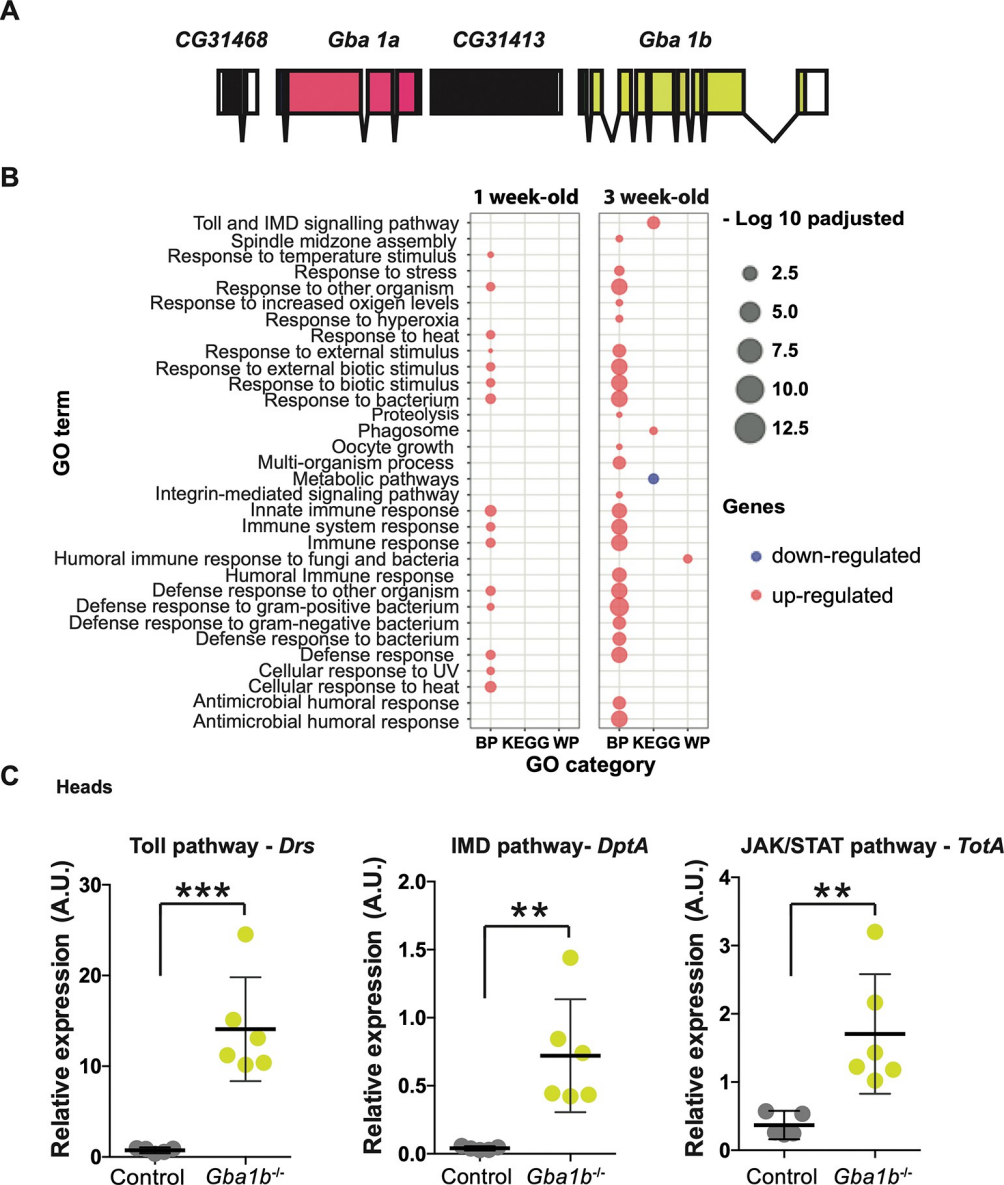
GCase deficiency results in up-regulation of innate immune pathways in the fly head. **(A)** Schematic representation of the two *Drosophila Gba1* gene loci, *Gba1a* and *Gba1b*. **(B)** Functional enrichment of the up-regulated and down-regulated genes in the heads of 1- and 3-week-old *Gba1b*^*-/-*^ flies relative to controls. All the significant GO-terms (adjusted p-values <0.05) for Biological Processes (BP), KEGG pathway (KEGG) and Wiki pathway (WP) are shown. There is strong up-regulation of GO-categories related to innate immune pathways. The size of the dots represents -log10 p-adjusted values for the GO-term enrichments. **(C)** Quantitative RT-PCR confirms up-regulation of the Toll (*Drs)* (***p = 0.0004), IMD (*DptA)* (**p = 0.0041) and JAK-STAT (*TotA*) (**p = 0.0069) reporter genes in 3-week-old *Gba1b*^*-/-*^ fly heads compared to controls. All target gene expression levels are normalized to tubulin. Unpaired t-tests; data are presented as mean ± 95% confidence intervals, n = 5–6 per genotype.

We have previously developed a *Drosophila* model of GCase deficiency by knocking out the two fly *Gba1* genes, *Gba1a* and *Gba1b* (Fig 1A), together or separately using homologous recombination (Kinghorn et al., 2016). Loss of *Gba1b* results in locomotor defects, reduced lifespan and neurodegeneration (Kinghorn et al., 2016). *Gba1b* displays widespread expression and is thus the predominant *GBA1* fly orthologue. It is significantly expressed in the nervous system and heart, with strongest expression levels in the fat body (FlyAtlas2) [19]. Several studies have identified that loss of *Gba1b*, but not *Gba1a*, recapitulates PD and GD phenotypes [20–23]. Thus, this study focused on flies lacking the *Gba1b* gene (*Gba1b*^*-/-*^).

To identify molecular and biological processes contributing to the observed phenotypes in *Gba1b*^*-/-*^ flies, we performed RNA-sequencing analysis on the heads of 1- and 3-week-old *Gba1b*^*-/-*^ and age-matched controls flies. This revealed that 207 genes were differentially expressed in *Gba1b*^*-/-*^ fly heads compared to controls at both time points, with greater differential gene expression at 3 weeks compared to 1 week (S1 Fig). Gene ontology analysis of both up- and down-regulated genes at both time points revealed significant gene enrichment for up-regulated genes mapping to pathways and biological processes related to the innate immune system, with greater up-regulation at 3 weeks (Fig 1B and S1 Fig). Many of these significantly expressed genes are key components of the Toll, IMD and JAK-STAT innate immune pathways (S1 Fig). We used quantitative RT-PCR (qRT-PCR) to independently examine innate immune gene expression. The AMP *Drosomycin* (*Drs*), a reporter gene of the Toll pathway, was significantly up-regulated in the heads of 3-week-old *Gba1b*^*-/-*^ flies compared to controls (Fig 1C). Similar changes were seen in the expression levels of *Diptericin A* (*DptA*), an AMP downstream of the IMD pathway and *TotA*, a reporter gene of the JAK-STAT pathway (Fig 1C). A similar increase in *DptA* and *TotA* gene expression, but not in *Drs* levels, was seen in the headless bodies of aged *Gba1b*^*-/-*^ flies, confirming peripheral innate immune activation (S2 Fig).

### GCase deficiency leads to brain glial activation and immune up-regulation in the fat body and gut

**Fig 2:**
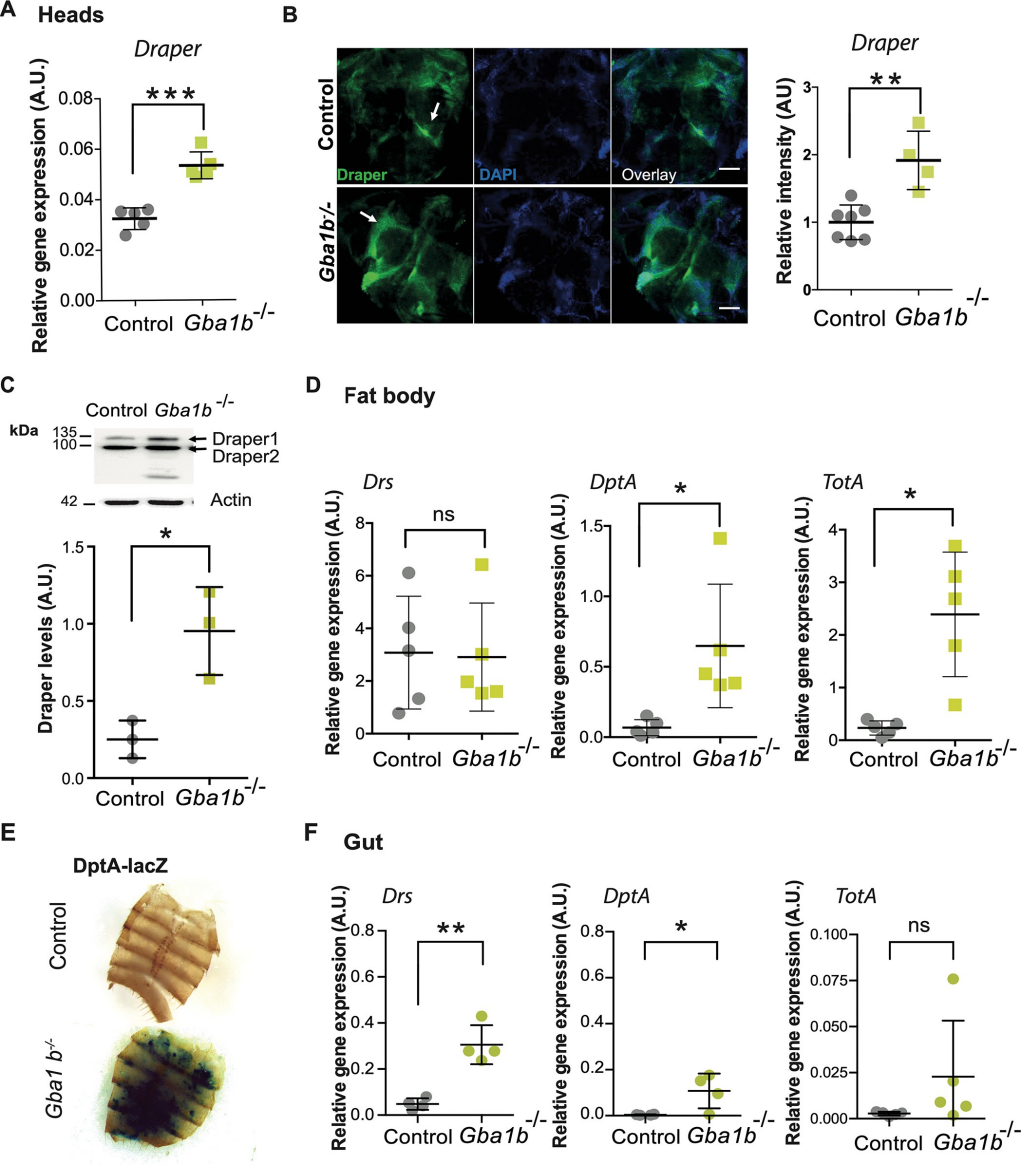
*Gba1b*^*-/-*^ flies display glial activation in the brain and immune activation in the fat body and gut. **(A)** Quantitative RT-PCR analysis demonstrates increased *Draper* gene expression in the heads of 3-week-old *Gba1b*^-/-^ flies compared to controls (***p = 0.0001). Unpaired t-test; data are presented as mean ± SD, n = 5 per genotype. **(B)** Draper immunofluorescence (green channel) is increased in 3-week-old *Gba1b*^-/-^ fly brains compared to age-matched controls (**p = 0.0015). White arrows show Draper localization in the antennal lobes. Scale bars, 50 μm. Representative images are shown. Unpaired t-test; data are presented as mean ± SD, n = 4–7 per genotype. **(C)** Western blot analysis confirms increased Draper 1 protein levels in the heads of *Gba1b*^-/-^ flies compared to controls. Draper levels are shown normalised to actin. Unpaired t-test (*p = 0.0354) and data presented as mean ± SD, n = 3 biological repeats per genotype. **(D)** Quantitative RT-PCR analysis confirms up-regulation of the IMD (*DptA)* (*p = 0.041) and JAK-STAT (*TotA*) (*p = 0.0147) reporter genes in the pooled dissected fat bodies of 3-week-old *Gba1b*^*-/-*^ flies compared to controls. All target gene expression levels are normalized to tubulin. Unpaired t-tests; data are presented as mean ± SD, n = 5 per genotype. **(E)** Expression of the IMD reporter, DptA-LacZ, in CRIMIC inserted *Gba1b* null flies reveals strong LacZ staining in the dissected fat body tissue. Representative images are shown. **(F)** The expression of the Toll innate immune pathway reporter gene, *Drs*, is increased in the midgut of 3-week-old *Gba1b*^*-/-*^ flies compared to controls (**p = 0.0011). The IMD (*DptA)* reporter gene is also significantly increased (*p = 0.0334), while the JAK-STAT reporter *TotA* is not significantly elevated (p = 0.1801). All target gene expression levels are normalized to tubulin. Unpaired t-tests; and data are presented as mean ± SD, n = 4–5 per genotype.

Since local innate immune pathways are activated in the heads and bodies of *Gba1b*^*-/-*^ flies, we studied the spatial pattern of the immune responses in more detail. Given the presence of immune-responsive fat body tissue within the head, evidence of neuroinflammation was probed in the *Gba1b*^*-/-*^ fly brain by examining the highly conserved Draper-dependent glial immune pathway. Draper is a phagocytic recognition receptor on the surface of engulfing glia in flies and is required for the phagocytic removal of cellular debris following axonal injury [24, 25]. Consistent with the widespread innate immune activation, we observed increased *Draper* gene expression in the heads of 3-week-old *Gba1b*^*-/-*^ flies (Fig 2A). Draper protein levels were also elevated in the *Gba1b*^*-/-*^ fly brain on immunostaining (Fig 2B) and on western blot analysis of fly heads (Fig 2C).

We next assessed the innate immune responses in the fat body, the main site of AMP production. In keeping with IMD and JAK-STAT pathway up-regulation in the bodies of *Gba1b*^*-/-*^ flies, there was a significant increase in *DptA* and *TotA* gene expression in dissected fat body tissue (Fig 2D). This was corroborated by the co-expression of an IMD reporter construct, DptA-LacZ. This revealed strong diffuse LacZ staining in the fat body tissue of flies lacking *Gba1b*, consistent with IMD pathway activation (Fig 2E). An increase in innate immune signalling in the intestinal tissue was also observed. *Drs* and *DptA* gene expression were significantly elevated in the midgut tissue of *Gba1b*^*-/-*^ flies compared to controls (Fig 2F). In addition, there was a trend towards up-regulation of *TotA* expression, although this did not reach statistical significance (Fig 2F). As *Gba1a* is predominantly expressed in the larval and adult fly midgut (FlyAtlas2) [19], we examined the innate immune responses in *Gba1a* knockout flies. Loss of *Gba1a* was not associated with any significant change in *Drs*, *DptA* or *TotA* expression in the heads, guts and headless bodies compared to controls (S3 Fig).

It has recently been demonstrated that *Gba1b* is expressed predominantly within glia, rather than neurons, in the fly brain [26]. Using the same CRIMIC *Gba1b* line, in which *Gba1b* gene expression is disrupted, we co-expressed UAS-mCherry.nls under the *Gba1b* endogenous promoter [27]. Fluorescent mCherry expression almost exclusively overlapped with the glial marker repo, with little overlap with the neuronal marker elav, confirming the glial-specific expression of *Gba1b* (S4A Fig). Interestingly, we also observed strong mCherry expression within the fat body and gut tissues (S4B and S4C Fig). Thus, the increased innate immune responses likely represent cell autonomous effects within the tissues where the *Gba1b* gene is expressed.

### *Gba1b* deficient flies exhibit increased intestinal transit time and gut wall permeability

**Fig 3:**
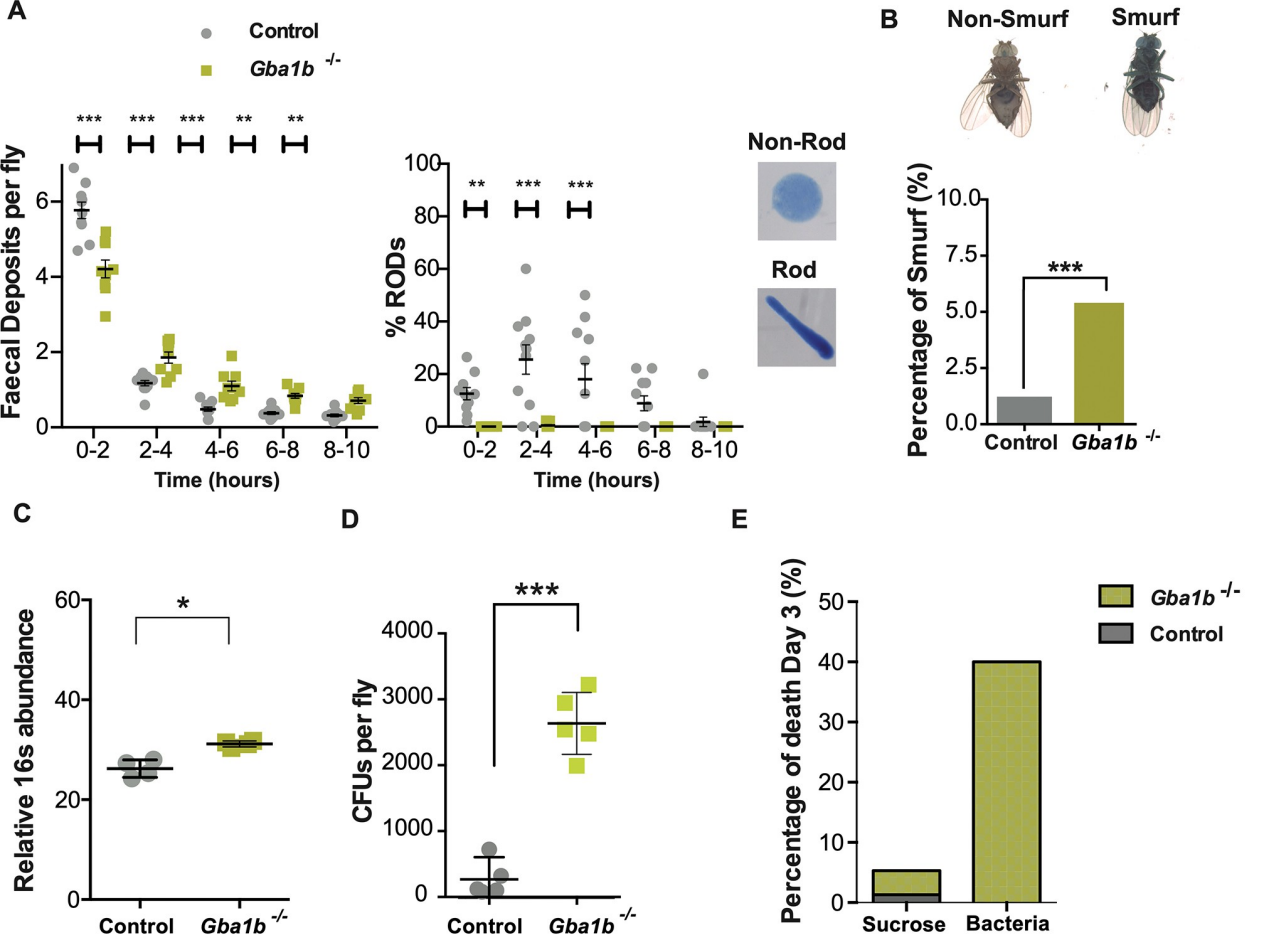
*Gba1b*^*-/-*^ flies show reduced intestinal transit and an altered gut microbiome. **(A)** The rate of intestinal transit is decreased in 3-week-old *Gba1b*^*-/-*^ flies compared to controls as assessed by the number of faecal deposits over time (***p< 0.0001 and ** p< 0.01). *Gba1b*^*-/-*^ flies produce almost exclusively non-ROD faecal deposits (***p< 0.0001 and ** p< 0.01). Two-way ANOVA followed by Fisher’s LSD multiple comparison test. Data are presented as mean ± SEM. **(B)** Assessment of gut permeability using a Smurf assay reveals that there is an increase in the number of Smurf flies among aged *Gba1b*^-/-^ flies (28 days-old) compared to controls, suggesting increased gut wall permeability (*Gba1b*^-/-^ vs control, ***p = 0.0002;). X^2^ (chi-squared) tests with Yates’ correction. **(C)** Quantitative PCR-based 16S rRNA gene abundance is significantly higher in the guts of *Gba1b*^*-/-*^ flies than in controls (*p = 0.0286). Mann-Whitney test; data are presented as mean ± SD, n = 4 per genotype. **(D)** CFUs are significantly increased in the guts of 3-week-old *Gba1b*^*-/-*^ flies compared to age-matched controls (p<0.001). Unpaired t-test; results are presented as mean ± SD, n = 5 per genotype). **(E)** Oral infection with *Lactobacillus plantarum* results in increased mortality among *Gba1b*^*-/-*^ flies but not in control flies.

The fact that PD patients exhibit intestinal inflammation and gastrointestinal (GI) abnormalities such as constipation, often preceding motor defects by several years [28], prompted us to investigate the intestinal physiology of *Gba1b*^-/-^ flies. Changes in intestinal function were identified by analysing the rate of faeces production following the feeding of food supplemented with 0.5% bromophenol blue (BPB). Aged *Gba1b*^*-/-*^ flies exhibited a delay in intestinal transit time, with significantly fewer faecal deposits produced in the first few hours after consuming BPB food compared to controls (Fig 3A). We also noted that the faecal content of control flies was more concentrated, with a high proportion of oblong shaped ROD deposits compared to non-ROD circular faecal deposits, whilst *Gba1b*^*-/-*^ flies almost exclusively produced non-ROD deposits (Fig 3A). The latter represent faeces with a higher water content [29]. Taken together, our results show delayed intestinal emptying and altered faecal content in *Gba1b*^*-/-*^ flies, in keeping with the gastrointestinal dysfunction seen in PD. The altered intestinal transit was not due to reduced feeding, as a CAFE assay revealed no significant difference in food consumption between aged *Gba1b*^*-/-*^ flies and age-matched control flies (S5B Fig).

In addition to GI dysfunction, compromised intestinal barrier integrity has also been reported in PD patients [30] [31]. We therefore employed a Smurf assay to assess gut permeability in *Gba1b*^*-/-*^ flies. This involved feeding with a non-absorbable blue food dye [32], and measuring the proportion of flies that display the presence of the blue dye outside of the gut, resulting in a blue Smurf appearance. Smurf flies were more abundant in the aged *Gba1b*^*-/-*^ population relative to age-matched controls, demonstrating compromised gut barrier integrity (Fig 3B). In addition to GI pathophysiological defects in PD patients, changes in the gut microbiome have been described as a precursor to neuropathology [33–35]. In view of the intestinal immune activation and gut dysfunction in *Gba1b*^-/-^ flies, we performed qPCR analysis using 16S universal primers and bacterial culture on midgut tissue from aged *Gba1b*^*-/-*^ flies and controls to assess microbial load. A significant increase in overall bacterial load by qPCR was observed in midguts from *Gba1b*^*-/-*^ flies compared to control flies (Fig 3C). This was supported by an increase in colony forming units (CFUs) in cultured gut extracts (Fig 3D). In addition, 16S ribosomal RNA-sequencing demonstrated clear differences in the intestinal microbes of *Gba1b*^*-/-*^ flies compared to control flies (S5A Fig). In particular, there was a significant increase in the *Acetobacter* and *Lactobacillus* genera in the gut of *Gba1b*^*-/-*^ flies.

Since *Gba1b*^*-/-*^ flies exhibit an increased bacterial load of altered composition, we determined if a bacterial challenge was a contributor to early mortality in *Gba1b*^*-/-*^ flies. We orally challenged *Gba1b*^*-/-*^ flies with the commensal bacteria *Lactobacillus plantarum* cultured from colonies isolated from control gut microbiota. Infected *Gba1b*^*-/-*^ flies displayed significantly greater mortality than control flies at day 3 post-infection (Fig 3E). Quantitative CFU assessment showed that there were no significant differences in the *Lactobacillus plantarum* gut load between *Gba1b*^*-/-*^ flies and controls after oral infection (S5C Fig). To further understand if the early mortality was due to bacterial proliferation or an overactivation of the innate immune response in the mutant flies, we determined the levels of *Drs* following an intrathoracic injection with the heat-killed bacteria *Staphylococcus aureus*. Aged *Gba1b*^*-/-*^ flies exhibited a ~3-fold change in *Drs* production compared to controls at 96 hours post injection (S5D Fig). Furthermore, intrathoracic injection with heat-killed *S*. *aureus* resulted in a reduction in the lifespan of *Gba1b*^*-/-*^ flies compared to flies injected with PBS vehicle alone (S5E Fig). The lifespan of control flies injected with heat-killed bacteria was not significantly different to that of PBS injected flies (S5E Fig). This suggests that it is the inflammatory stimulus and its uncontrolled immune response, rather than bacterial proliferation, that results in compromised survival in *Gba1b*^*-/-*^ flies. Together our data suggest that *Gba1b*^*-/-*^ flies are unable to effectively regulate and respond to commensal gut microbiota, contributing to adverse health outcomes.

### Amelioration of the gut microbiome under germ-free conditions partially rescues survival and locomotor phenotypes in *Gba1b*^*-/-*^ flies

**Fig 4:**
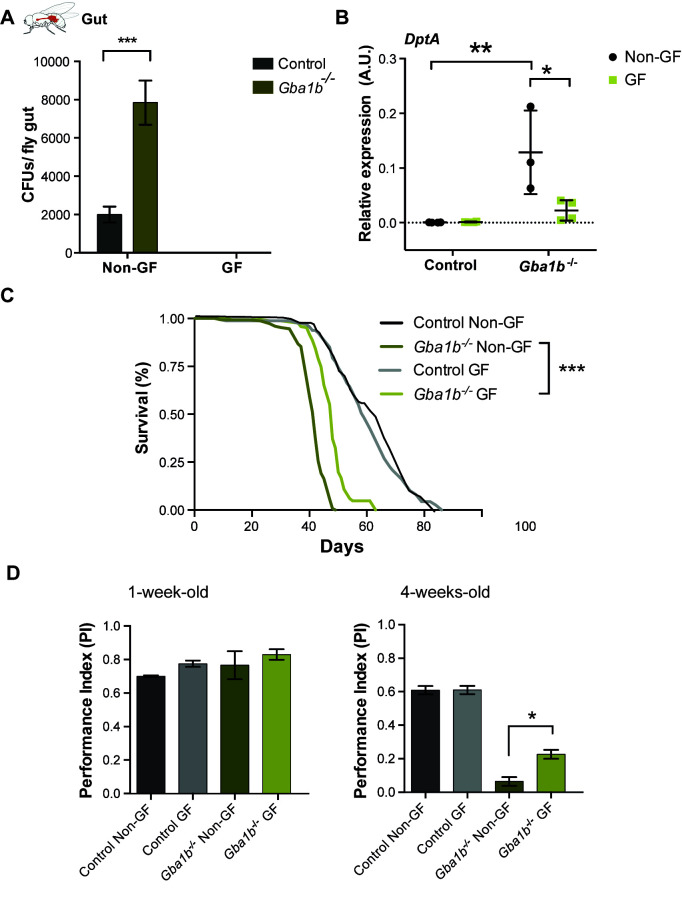
Raising *Gba1* mutant flies under germ-free (GF) conditions partially ameliorates a number of disease phenotypes. **(A)** CFUs in *Gba1b*^-/-^ and control fly guts raised under non-GF and GF conditions at 3 weeks demonstrates that *Gba1b*^-/-^ flies display higher microbial load in comparison with control flies (***p<0.0001). No bacterial load is observed for both control and mutant flies raised under GF conditions. Two-way ANOVA test followed by Tukey’s multiple comparison test. Data are presented as mean ± SEM, n = 5 per condition. **(B)** Quantitative RT-PCR analysis of *DptA* mRNA levels, in the gut of non-GF and GF *Gba1b*^-/-^ and control flies, shows *DptA* levels are significantly reduced in GF *Gba1b*^-/-^ flies (**p = 0.0027; *p = 0.0107). Two-way ANOVA test followed by Sidak’s multiple comparison test. Data are presented as mean ± SD, n = 3–4 per condition. **(C)** GF *Gba1b*^-/-^ treated flies have an increased lifespan compared to those reared under standard non-GF conditions. Log-rank tests were used for all comparisons: GF vs non-GF*Gba1b*^-/-^, p = 0.0003 (n = 150); GF control vs non-GF control p = 0.9813 (n = 150). **(D)** GF *Gba1b*^-/-^ flies have improved climbing ability at 4 weeks of age compared to their non-GF counterparts (ns > 0.05; *p = 0.034 GF vs non-GF *Gba1b*^-/-^). One-way ANOVA test with Tukey’s post hoc analysis (n = 75 flies per condition).

To test whether modulating the microbiome would have beneficial effects, *Gba1b*^*-/-*^ and control flies were reared under germ-free (GF) conditions and behavioural phenotypes were assessed. Adult control and mutant flies were raised and aged on standard food containing a cocktail of antibiotics to maintain GF conditions (Fig 4A). Raising *Gba1b*^*-/-*^ flies under GF conditions resulted in a significant reduction of *DptA* expression as well lifespan extension (Fig 4B and 4C). These survival benefits appeared to be specific to *Gba1b*^*-/-*^ flies, as GF control flies did not exhibit improved lifespan compared with non-GF controls. GF conditions also improved the locomotor ability of aged *Gba1b*^*-/-*^ flies compared to their non-GF counterparts (Fig 4D). Thus, modulation of the intestinal microbiome partially ameliorates lifespan and locomotor phenotypes in GCase-deficient flies.

### The gut microbiota regulates the systemic innate immune response in *Gba1b*^*-/-*^ flies

**Fig 5:**
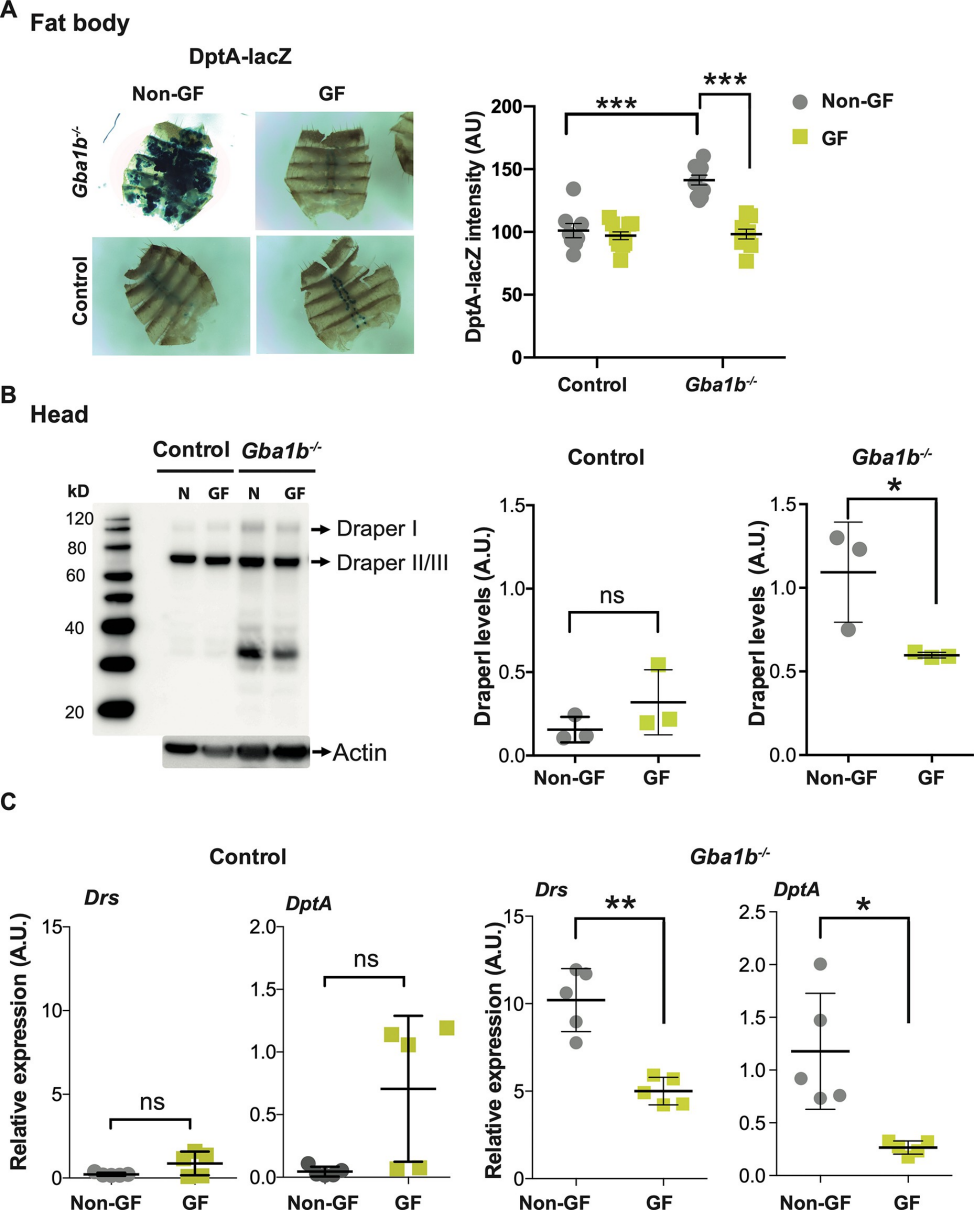
Raising *Gba1b*^*-/-*^ flies under GF conditions reverses immune activation in fat body and brain tissues. **(A)** DptA-LacZ staining in the fat body is reduced to control levels in GF *Gba1b*^*-/-*^ flies compared to their non-GF counterparts (***p<0.0001). One-way ANOVA and Tukey’s post hoc analysis; data are presented as mean ± 95% confidence intervals. **(B)** Western blot analysis reveals a decrease in Draper 1 levels in the heads of GF *Gba1b*^*-/-*^ flies compared to non-GF flies (n = 3; GF *Gba1b*^*-/-*^ vs non-GF *p = 0.045; GF controls vs non-GF p = 0.2472). **(C)** *DptA* and *Drs* transcript levels are reduced on qRT-PCR analysis of heads of 3-week-old GF *Gba1b*^*-/-*^ flies compared to non-GF flies (*p = 0.0202; **p = 0.0014). Unpaired t-test. Data are presented as mean ± SD (n = 5 per condition).

The effect of eliminating the intestinal microbiome on immune activation in the fat body and head was also examined. *Gba1b*^*-/-*^ flies exhibited a striking reduction in fat body DptA-LacZ staining towards control levels when raised under GF conditions (Fig 5A). Additionally, raising *Gba1b*^*-/-*^ flies under GF conditions resulted in decreased glial activation, as evidenced by a reduction in Draper protein levels in the head (Fig 5B). Remarkably, GF *Gba1b*^*-/-*^ flies displayed a significant reduction in *DptA*, *Drs*, and other innate immune markers such as *Attacin A* (*AttA*), *Peptidoglycan recognition protein-Sc2* (PGRP*-SC2*), *Cecropin C* (*CecC*) and *Drosocin* (*Dro*), indicative of reduced innate immune signaling (Fig 5C and S6 Fig). Together, our data suggest that the gut microbiota is responsible for immune activation in the gut and other tissues of GCase-deficient flies, and that reversal of this microbiome-dependent immune activation is associated with partial amelioration of lifespan and neuromuscular phenotypes.

### Overactivation or deletion of the IMD pathway is detrimental to flies lacking *Gba1b*

**Fig 6:**
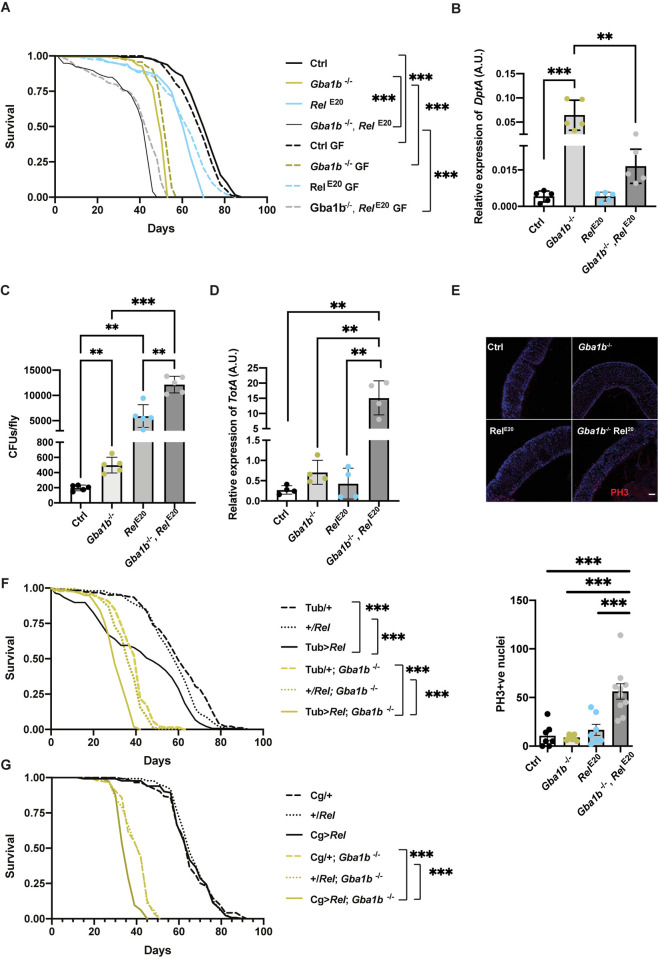
Overexpression or knockout of *Rel* is toxic to flies lacking *Gba1b*. **(A)** The lifespan of *Gba1b*^-/-^, *Rel*^E20^ flies is significantly reduced under both GF and non-GF conditions when compared to single *Gba1b*^-/-^ and *Rel*^E20^ mutants. Log-rank tests were used for all the comparisons (n = 150), in non-GF: Ctrl vs *Gba1b*^-/-^ ***p = 1.2x10^-49^; Ctrl vs *Rel*^E20^ ***p = 2.3x10^-24^; *Gba1b*^-/-^ vs *Gba1b*^-/-^, *Rel*^E20^ ***p = 1.9x10^-40^ and in GF vs non-GF: Ctrl *p = 0.02; *Gba1b*^-/-^ ***p = 8.24x10^-10^; *Gba1b*^-/-^, *Rel*^E20^ double mutant ***p = 1.22x10^-6^. **(B)** *DptA* transcript levels are reduced on qRT-PCR analysis of headless bodies of 3-week-old *Gba1b*^*-/-*^, *Rel*^E20^ flies (n = 4–5 per condition; ***p = 0.0001 and **p = 0.0011). One-way ANOVA followed my multiple comparison tests. Data are presented as mean ± SD. **(C)** 3-week-old *Gba1b*^-/-^, *Rel*^E20^ flies display higher microbial load. Ctrl vs *Gba1b*^*-/-*^ **p = 0.0017; Ctrl vs *Rel*^E20^ **p = 0.0047; Ctrl vs *Gba1b*^-/-^, *Rel*^E20^ ***p<0.0001; *Gba1b*^-/-^ vs *Gba1b*^-/-^, *Rel*^E20^ ***p<0.0001; *Rel*^E20^ vs *Gba1b*^-/-^, *Rel*^E20^ **p = 0.0013). One-way ANOVA test followed by multiple comparison test; data are presented as mean ± SD. **(D)** *TotA* transcript levels are increased on qRT-PCR analysis of 3-week-old headless bodies of *Gba1b*, *Rel*^E20^ flies (n = 4–5 per condition; Ctrl vs *Gba1b*^*-/-*^, *Rel*^E20^ **p = 0.0023; *Rel*^E20^ vs *Gba1b*^*-/-*^, *Rel*^E20^ **p = 0.0025; and *Gba1b*^*-/-*^ vs *Gba1b*^*-/-*^, *Rel*^E20^ **p = 0.0035). One-way ANOVA followed by Fisher’s multiple comparison tests; data are presented as mean ± SD. **(E)** Gut immunostaining for PH3 demonstrates a greater number of PH3 positive cells in the midgut of 3-week-old *Gba1b*^*-/-*^, *Rel*^E20^ flies (*** p<0.0001). One-way ANOVA followed by multiple comparison tests; data are presented as mean ± SD. Scale bar = 50μm. **(F)** Ubiquitous overexpression of *Rel* using Tubulin-GAL4 (Tub) driver is deleterious to *Gba1b*^*-/-*^ flies. Log-rank tests were used for all the comparisons (n = 150): +/Tub; *Gba1b*^*-/-*^ vs Tub>*Rel*; *Gba1b*^*-/-*^ ***p = 1.69x10^-24^ and +/*Rel*; *Gba1b*^*-/-*^ vs Tub>*Rel*; *Gba1b*^*-/-*^ ***p = 8.68x10^-16^, +/Tub; *Gba1b*^*-/-*^ vs +/*Rel*; *Gba1b*^*-/-*^ * p = 0.045, +/*Rel* vs Tub>*Rel* **p = 0.003 and +/Tub vs Tub>*Rel* ***p = 1.22x10^-11^. **(G)** Overexpression of *Rel* in the fat body using the Cg-GAL4 (Cg) driver is deleterious to *Gba1b*^*-/-*^ flies. Log-rank tests were used for all the comparisons (n = 150): +/Cg; *Gba1b*^*-/-*^ vs Cg>*Rel*; *Gba1b*^*-/-*^ ***p = 4.76x10^-17^ and +/*Rel*; *Gba1b*^*-/-*^ vs Cg>*Rel*; *Gba1b*^*-/-*^ ***p = 2.56x10^-15^, +/Cg; *Gba1b*^*-/-*^ vs +/*Rel*; *Gba1b*^*-/-*^ p = 0.9 and +/Cg vs Cg> *Rel* p = 0.70.

Following the observation that GF conditions reduce the systemic immune response in *Gba1b*^*-/-*^ flies, with improvements in survival and locomotor ability, we proceeded to investigate the effect of genetically manipulating the IMD pathway. A double knockout of both *Relish* (*Rel*), a core NF-*k*B transcription factor of the IMD pathway, and *Gba1b* was generated (*Gba1b*^-/-^, *Rel*^E20^). The lifespan of *Gba1b*^-/-^, *Rel*^E20^ flies raised under both non-GF and GF conditions was decreased compared to both *Rel*^E20^ and *Gba1b*^*-/-*^ single mutants (Fig 6A), suggesting that IMD pathway signaling is beneficial to *Gba1b*^*-/-*^ flies. As expected, *Gba1b*^-/-^, *Rel*^E20^ flies showed a significant decrease in *DptA*, demonstrating that the *Rel*^E20^ mutant abrogates IMD pathway activation (Fig 6B). *Drs* was similarly decreased, suggesting there is no compensatory activation of the Toll pathway in the double mutant (S7A Fig).

To identify a possible cause for the increased mortality of *Gba1b*^-/-^, *Rel*^E20^ flies, we analysed the gut microbial load. Knockout of *Rel* resulted in a dramatic increase in bacterial load, which was further increased in *Gba1b*^-/-^, *Rel*^E20^ flies (Fig 6C). In addition to the increased microbial load, significantly higher levels of *TotA* were detected in *Gba1b*^-/-^, *Rel*^E20^ flies (Fig 6D), suggesting an enhanced activation of the JAK/STAT damage response pathway. These results led us to hypothesise that an elevated bacterial count in the gut might induce damage and increased turnover of the intestinal epithelium. To assess this, we examined the proliferation of the intestinal stem cells (ISC) by immunostaining the guts of both controls and *Gba1b*^-/-^, *Rel*^E20^ flies using the mitotic marker PH3. Notably, we observed an increase in ISC proliferation within the guts of *Gba1b*^-/-^, *Rel*^E20^ double mutant flies (Fig 6E).

To assess whether the high die-off in *Gba1b*^-/-^ flies, following exposure to the commensal bacteria *Lactobacillus plantarum*, was the result of IMD immunotoxicity, we examined the response of *Gba1b*^-/-^, *Rel*^E20^ double mutants to bacterial feeding. Consumption of *Lactobacillus plantarum* led to a substantial increase (~70%) in fly mortality within 5 days among aged *Gba1b*^-/-^, *Rel*^E20^ double mutants, whereas it caused a more moderate (~20%) mortality among the single *Gba1b*^-/-^ and *Rel*^E20^ mutant flies (S7B Fig). Raising *Gba1b*^-/-^, *Rel*^E20^ flies under GF conditions partially ameliorated the toxic lifespan effect (Fig 6A). Collectively, these results demonstrate the indispensable role of the IMD/Rel pathway in regulating microbial load and maintaining intestinal epithelial homeostasis in *Gba1b*^*-/-*^ flies.

To test whether IMD activation is immunotoxic, we overexpressed *Rel* both ubiquitously and in the fat body of *Gba1b*^*-/-*^ flies. This resulted in increased mortality (Fig 6F and 6G), demonstrating that augmentation of IMD immune signalling is detrimental in a *Gba1b* deficient background. Increased *Rel* expression was also confirmed using qRT-PCR (S7D Fig). Moreover, the level of *TotA* gene expression was also increased in *Gba1b*^*-/-*^ flies overexpressing *Rel* (S7E Fig), suggesting activation of the JAK-STAT stress response. Together, these findings indicate that IMD/Rel pathway activation needs to be fine-tuned in *Gba1b* deficiency. Rel appears to play a pivotal protective role in regulating *Gba1b*^-/-^ microbial loads but is immunotoxic when overexpressed.

### Partial amelioration of the autophagy impairment in *Gba1b*^-/-^ flies leads to reduced IMD signaling

**Fig 7:**
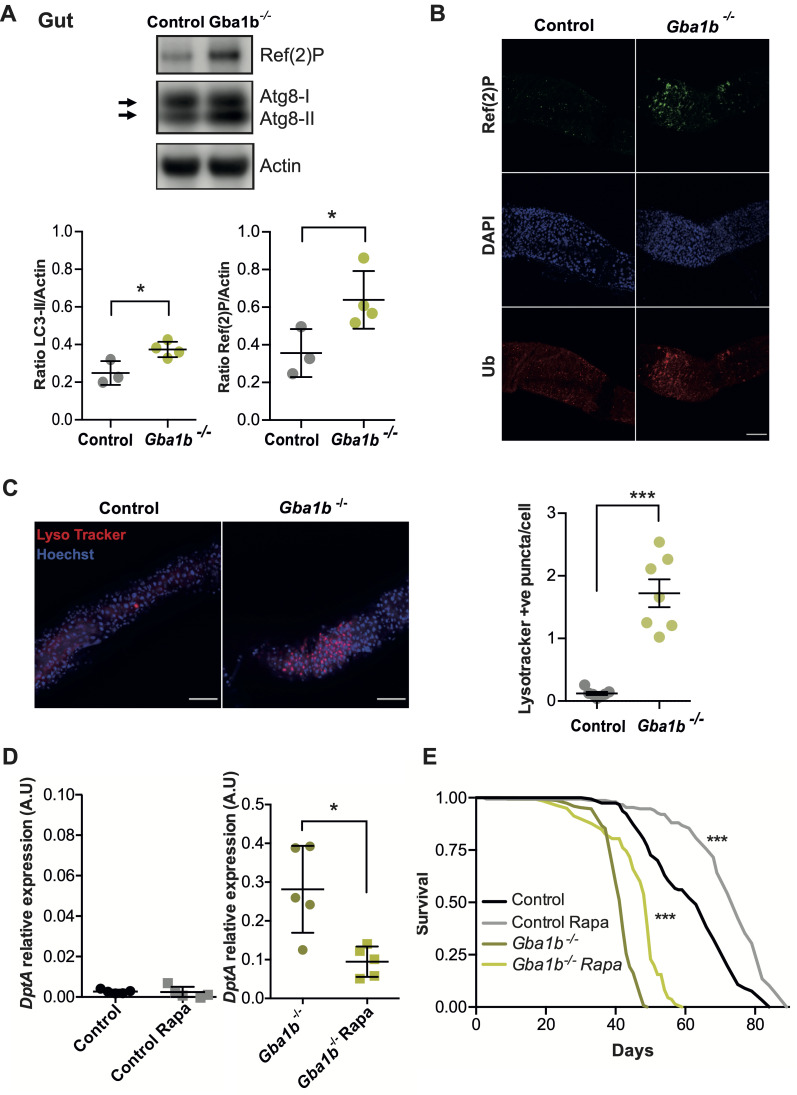
Loss of *Gba1b* results in gut autophagy impairment and administration of rapamycin rescues *Gba1b*^*-/-*^ immune phenotypes. **(A)** *Gba1b*^*-/-*^ fly guts show significant accumulation of Atg8a-II and Ref(2)P proteins relative to control guts (*p = 0.024 and *p = 0.049, respectively). Unpaired t-tests; data are presented as mean ± SD (n = 3–4). (B) Immunostainings of guts labelled for Ref(2)P (green), DAPI (blue) and ubiquitin (Ub, red). *Gba1b*^*-/-*^ flies display a higher number of aggregates of Ref(2)P and ubiquitinated proteins in the midgut. Scale bar 100 μm. (C) Gut staining with LysoTracker (red) and Hoechst (blue) of 3-week-old flies reveals an increased number of LysoTracker puncta in *Gba1b*^*-/-*^ flies (***p<0.0001; unpaired t-test). Scale bar 50 μm. (D) *DptA* transcript levels are reduced on qRT-PCR analysis of the guts of 3-week-old *Gba1b*^*-/-*^ flies treated with Rapamycin (Rapa) compared to non-treated flies (*p = 0.0159). No significant differences are found in control flies (ns = 0.5317). Mann Whitney tests; data are presented as mean ± SD (n = 5 per condition). (E) The survival of Rapa treated control and *Gba1b*^-/-^ flies (n = 150) is significantly increased. Log-rank tests: *Gba1b*^-/-^ vs *Gba1b*^-/-^ Rapa, *** p<0.0001; Control vs Control Rapa *** p<0.0001.

Autophagy has been shown to have a role in dampening innate immune responses and is achieved by removing key signal transduction components from these pathways [36–39]. Loss of *Gba1b* has previously been shown to disrupt autophagic flux in the brain in an age-dependent manner [22]. As the nexus point of microbe-host interactions, autophagy was assessed in the gut of *Gba1b*^*-/-*^ flies. For this purpose the accumulation of Ref(2)P, the fly ortholog of the selective autophagosomal receptor P62, and Atg8a, the fly ortholog of LC3, were used as markers of selective autophagy and macroautophagy respectively [40]. Western blot analysis on dissected fly guts revealed that both Atg8a-II and Ref(2)P protein levels were significantly increased in *Gba1b*^*-/-*^ flies when compared with control flies (Fig 7A). A significant increase in the number and size of Ref(2)P and ubiquitin (Ub) puncta was seen on immunostaining in *Gba1b*^*-/-*^ flies compared to control fly guts (Fig 7B). We observed several distinct LysoTracker-positive puncta within the midgut region of 3-week-old *Gba1b*^*-/-*^ flies, but not in control fly guts (Fig 7C), which is characteristic of impaired autophagic turnover [41, 42]. Overall, our data mirrors the autophagy defects seen in the brains of *Gba1b*^*-/-*^ flies [22].

To investigate whether the abnormal immune activation that we observe in *Gba1b*^*-/-*^ flies may result from a loss of autophagy-mediated regulation of immune signaling, autophagy was induced by raising flies on food supplemented with rapamycin a well-known inhibitor of Torc1. Chronic rapamycin treatment resulted in increased levels of LysoTracker-positive puncta in both control and mutant guts and higher ratios of Atg8a-II/I, indicative of increased autophagic flux (S8 Fig). Importantly, rapamycin treatment significantly reduced the levels of the IMD pathway reporter *DptA* in the gut and extended the lifespan of *Gba1b*^*-/-*^ flies (Fig 7D and 7E).

### The beneficial effects of rapamycin treatment are not enhanced under germ-free conditions in *Gba1b^-/-^* flies

**Fig 8:**
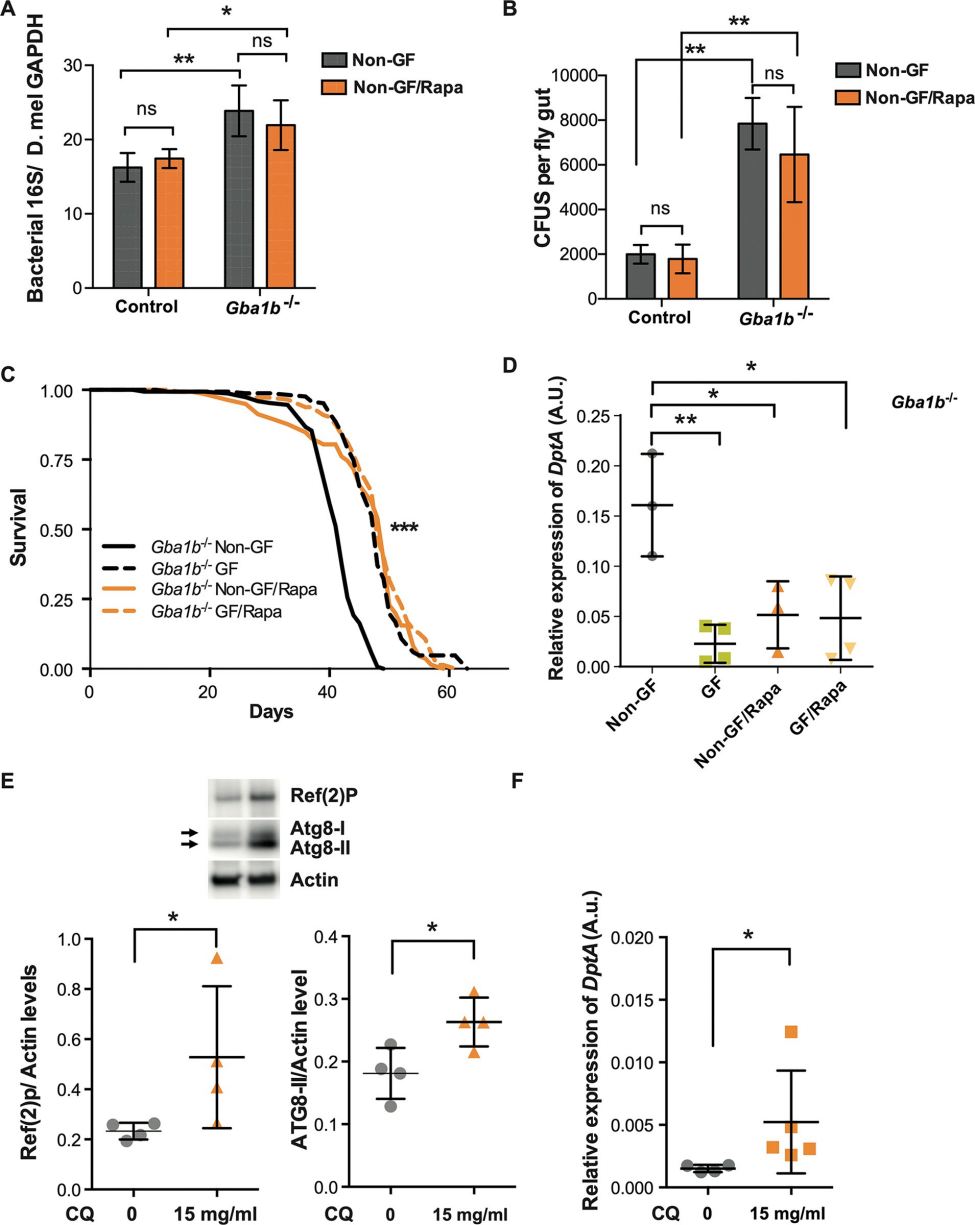
Long-term rapamycin treatment improves *Gba1b*^*-/-*^ fly survival by reducing inflammation without altering microbial load. **(A)** Quantitative PCR using primers for 16S rRNA gene on the guts of 3-week-old flies does not reveal significant differences in bacterial load of Rapa treated flies for the two genotypes. Significant differences in the bacterial load are observed in control vs *Gba1b*^*-/-*^ non-GF flies (**p = 0.0077) and control vs *Gba1b*^*-/-*^ non-GF flies treated with Rapa (*p = 0.045). Two-way ANOVA followed by Tukey’s multiple comparison test; data are presented as mean ± SD (n = 4 per condition). (B) There are no significant differences in the CFUs of 3-week-old guts of non-GF flies treated or not treated with Rapa (control ns = 0.991; *Gba1b*^*-/-*^ ns = 0.57). Significant differences were found in the comparisons control vs *Gba1b*^*-/-*^ non-GF flies (**p = 0.0022) and control vs *Gba1b*^*-/-*^ non-GF flies treated with Rapa (**p = 0.0087). Two-way ANOVA followed by Tukey’s multiple comparison tests; data are presented as mean ± SD. (C) GF and Rapa individual treatments extend the lifespan of *Gba1b*^*-/-*^ flies to a similar extent (***p<0.0001). No significant additive lifespan extension is observed in GF *Gba1b*^*-/-*^ flies treated with Rapa. Log rank test; n = 150. (D) Quantitative RT-PCR analysis of *DptA* transcript levels in guts from *Gba1b*^*-/-*^ flies raised under GF, Rapa and GF/Rapa conditions demonstrates no additive effect on the lowering of *DptA* levels (non-GF vs GF, **p = 0.0030; non-GF vs non-GF/Rapa, *p = 0.0207; non-GF vs GF/Rapa, *p = 0.0117; GF vs non-GF/Rapa, p = 0.7434; GF vs GF/Rapa, p = 0.766; non-GF/Rapa vs GF/Rapa, p = 0.999). One-way ANOVA followed by Tukey’s multiple comparison test. Data presented as mean ± SD (n = 3–4). (E) Western blot analysis of Ref(2)P and Atg8a on the guts from 3-week-old control flies treated with chloroquine (CQ) for 48 hours. CQ treatment significantly induces accumulation of Ref(2)P (*p = 0.028) and Atg8a-II (*p = 0.0269). Mann Whitney tests; data are presented as mean ± SD (n = 4 per condition). (F) *DptA* transcript levels are increased on qRT-PCR analysis of 3-week-old control fly guts treated with CQ for 48 hours compared to non-treated flies (*p = 0.0159; Mann Whitney test; n = 4 per condition). Data are presented as mean ± SD.

Rapamycin treatment has been shown to reduce bacterial load in aged flies [43, 44], raising the possibility that the rapamycin-mediated lifespan improvement in *Gba1b*^*-/-*^ flies was dependent upon the presence of the intestinal microbiota. To study if this was the case, the bacterial load in the guts of rapamycin treated and non-treated flies was measured by qPCR for the 16S rRNA gene and quantifying bacteria CFUs. Rapamycin treated *Gba1b*^*-/-*^ and control flies exhibited similar expression of 16S rRNA and CFUs to non-treated flies (Fig 8A and 8B). Thus, rapamycin is neither directly bactericidal, nor is its effect as an IMD pathway suppressor contingent on a decrease in gut bacteria.

To directly determine whether rapamycin and GF treatment exert independent effects, GF and non-GF *Gba1b*^-/-^ flies were treated with rapamycin or vehicle control (EtOH). Rapamycin similarly extended the lifespan of mutants under GF and non-GF conditions (Fig 8C). No additive effect was observed when flies were treated with rapamycin in the absence of the microbiota, suggesting both treatments are partially rescuing the fly survival through a common downstream mechanism. As rapamycin treatment reduces *DptA* levels, we tested the effect of both interventions on the AMP levels of *Gba1b*^-/-^ flies. In agreement with the effect on lifespan, we found that GF *Gba1b*^*-/-*^ flies treated with rapamycin had similar levels of *DptA* when compared with non-GF *Gba1b*^-/-^ rapamycin treated flies (Fig 8D). To exclude the possibility that elimination of the microbiome may be exerting its beneficial effects via modulation of autophagy, the levels of Ref(2)P and Atg8a were shown to be unaltered in GF *Gba1b*^-/-^ flies versus their non-GF counterparts (S6B Fig). There were also no significant differences in LysoTracker staining and Ref(2)P immunostaining in *Gba1b*^*-/-*^ fly brains upon GF treatment (S6C Fig). Together, our results indicate that reduction of the innate immune response, either by improving intestinal autophagic degradation of putative immune cascade components or elimination of the microbiota, is sufficient to improve the lifespan of the *Gba1b*^*-/-*^ flies.

Finally, to further substantiate the link between defective intestinal autophagy and persistent activation of the innate immune response, we inhibited late-stage autophagy in control flies to mimic the autophagosome-lysosome fusion that occurs in GCase deficiency [45]. For that purpose, we fed flies with chloroquine (CQ), a well-characterized autophagy inhibitor that blocks autolysosome fusion [46], and then measured the effect on the autophagic and immune pathways. Feeding flies with CQ resulted in accumulation of Atg8a-II and Ref(2)P (Fig 8E) and significantly increased *DptA* expression in the gut tissue (Fig 8F). Overall, these data suggest that basal autophagy is intricately involved in the control of the innate immune response, and that blockage of autophagy in the gut is sufficient to recapitulate the immune phenotypes observed in *Gba1b*^-/-^ fly guts.

## Discussion

The immune system and gut-brain axis communication are increasingly being implicated in neurodegenerative disease. Using a GCase loss-of-function fly model of GD, we demonstrated that an altered gut microbiota, coupled with intestinal autophagic dysfunction, directly stimulates innate immune activation, in association with reduced lifespan.

Our genome-wide genetic analysis revealed that loss of GCase activity leads to an age-dependent increase in the expression of Toll, IMD and JAK-STAT pathway components. The *GBA1* gene is widely expressed in the human brain (proteinatlas.org) [47] and shows significantly higher expression in astrocytes and microglial/macrophage cells compared to neurons (brainrnaseq.org). Moreover, here we showed increased Draper-dependent glial activation in the *Gba1b*^*-/-*^ fly brain. Microglia, the dominant innate immune cells of the mammalian brain, are increasingly being implicated in neurodegenerative disease [48]. Reactive microglia are present in post-mortem PD brains [49] and positron emission studies have similarly reported increased microglial responses in cortical and subcortical areas *in vivo* in early PD, as well as in the brains of *GBA1* mutation carriers without PD [50–52]. Mouse models of neuronopathic GD have also revealed widespread astrogliosis and microgliosis, in association with fatal neurodegeneration within the first weeks of life [53, 54].

In addition to innate immune activation in the fly head, we confirmed that *Gba1b*^*-/-*^ flies display elevated peripheral innate immune responses, including in the gut and fat body. This is in keeping with a previous study in flies harbouring insertions of minos elements in the two *Gba1* genes, resulting in the production of truncated mutant GCase proteins. These flies displayed up-regulation of IMD and Toll pathway genes in the head and body [20, 55]. Our results thus confirm that complete loss of *Gba1b* gene activity is associated with strong innate immune activation, including the JAK-STAT stress response. Our finding that innate immune activation occurs peripherally is also consistent with studies showing evidence of significantly higher levels of serum proinflammatory cytokines and altered immune cell profiles in the blood of PD and GD patients compared to heathy individuals [56, 57].

There is increasing evidence for intestinal dysfunction in PD [28, 31], but it is not known whether gut pathology is a feature of *GBA1*-PD or GD. We demonstrated gut dysfunction with increased intestinal transit time and elevated gut wall permeability in *Gba1b*^*-/-*^ flies. Further probing revealed increased innate immune activation, predominantly of the IMD and Toll pathways, in the intestinal tissue of *Gba1b*^*-/-*^ flies. It is likely that these gut pathologies are a consequence of GCase loss-of-function in the intestinal tissue, as we confirmed *Gba1b* gene expression in the *Drosophila* gut. Importantly, our findings reflect those seen in PD patients where enteric inflammation on colonic biopsies has been reported, including increased intestinal expression of pro-inflammatory cytokines such as TNF-*α*, IL6 and IL-1*β*, and the bacterial endotoxin ligand TLR4 [28, 58]. Furthermore, intestinal permeability is increased in PD patients in association with decreased serum LPS binding protein (LBP), indicating greater endotoxin exposure [30, 59].

The intestinal microbiota is essential for the management of gut epithelial integrity, through maintenance of tight junction proteins and mucin production, thereby inhibiting the infiltration of bacteria and immunogenic products [60]. The *Drosophila* midgut is an ideal model system for studying host-microbiome interactions due to the striking conservation of intestinal structure and gut immune signaling with humans [61]. Consistent with the intestinal microbiome playing a role in *GBA1*-associated neurodegeneration, we observed increased intestinal bacterial load and alterations in the composition of the gut microbiome in *Gba1b*^*-/-*^ flies. There was a significant increase in the abundance of *Acetobacter* and *Lactobacillus*, the commonest genera in the fly gut [62]. A recent study has demonstrated that GluCer positively regulates growth of certain bacterial species [63], so GluCer deposition in intestinal cells of the gut lumen may trigger a shift in bacterial number and composition.

The fact that bacterial load was increased in *Gba1b*^*-/-*^ flies compared to control flies is commensurate with the observation that microbial load is a significant determinant of lifespan in *Drosophila* [64]. Our results also mirror several studies reporting changes in several gut bacterial species between PD patients and control groups [35, 65, 66]. The chronic gut immune activation observed in *Gba1b*^*-/-*^ flies likely promotes further microbiome dysbiosis, which in turn can lead to impairment of gut function and ultimately reduced survival [67]. Support for intestinal microbiome-mediated effects on the CNS in *Gba1*^-/-^flies, i.e., via gut-brain axis communication, was achieved by raising mutant and control flies under GF conditions. Modulation of the intestinal microbiome in GF flies resulted in partial amelioration of a number of phenotypes, including survival, locomotor defects and glial activation. Similar beneficial effects were seen on modulation of the intestinal microbiota in Alzheimer’s disease mouse and fly models [68–70] and α-Syn overexpressing (ASO) mice [71]. In the case of ASO mice, amelioration of the intestinal microbiome led to an improvement in neuropathology, whereas oral administration of specific microbial metabolites to GF mice promoted neuroinflammation and motor deficits. Interestingly, we show that the re-introduction of a single commensal bacteria from the gut of healthy control flies is sufficient to promote immune activation in *Gba1b*^*-/-*^ flies in association with significant mortality.

The finding that the intestinal microbiome in *Gba1b*^*-/-*^ flies is necessary to promote innate immune activation can be interpreted in the context of the recently described endotoxin hypothesis of neurodegeneration [72]. Endotoxins predominantly refer to the lipopolysaccharide found in the outer cell wall of gram-negative bacteria [62]. We hypothesise that the intestinal inflammation and altered gut microbiome observed in our *Gba1b*^*-/-*^ fly model promotes intestinal barrier permeability (‘gut leakiness’). This in turn allows translocation of PAMPs—among them endotoxins and peptidoglycan fragments—from the gut lumen into the systemic circulation [59, 72]. The increased intestinal transit time and increased bacterial load observed in *Gba1b*^*-/-*^ flies may further promote this translocation due to the increased exposure of the gut wall to bacteria. The subsequent circulating microbial products can stimulate a chronic systemic innate immune activation [73], consequently leading to neuroinflammation [58, 74, 75].

Furthermore, we showed that blockage of autophagy in healthy flies is sufficient to induce an immune response in the gut. This is in agreement with recent studies that show degradation of IMD pathway mediators, such as Tak1/Tab2 and IKK complex is mediated by selective autophagy. Loss of normal autophagic flux results in constitutive activation of the IMD pathway [38, 39]. Thus, the autophagy impairment observed in the brain, gut and fat body tissue of *Gba1b*^*-/-*^ flies may lead to an inability to terminate activated immune responses, through the degradation of key components of the NF-*k*B signalling cascades. In turn, this would lead to increased levels of AMPs. We therefore propose that the deleterious chronic inflammation observed in *Gba1b*^*-/-*^ flies results from the loss of autophagic regulation of immune signal transduction pathways.

Interestingly, despite the beneficial effects of eradicating the immune-stimulating gut microbiome, direct manipulation of IMD signaling did not produce similar benefits. Indeed, both knockout and overexpression of *Rel* was detrimental to *Gba1b* deficient flies. The survival of *Gba1b*^-/-^, *Rel*^E20^ double mutants was significantly reduced under both GF and non-GF conditions, demonstrating that chronic suppression of IMD signalling is toxic to *Gba1b*^-/-^ flies. Our data also indicate that gut bacterial load is likely partially contributing to the observed shortened lifespan, since *Rel* knockout in *Gba1b*^-/-^ flies is less toxic under GF conditions. Ubiquitous and fat body overexpression of *Rel* in *Gba1b*^-/-^ flies also reduces fly survival. Together these findings suggest that immune signaling requires fine-tuning in the context of *Gba1b* deficiency. In addition, studies have shown immune independent roles for *Rel* [76, 77]. These include regulation of the autophagy protein Atg1. It is therefore possible that dysregulation of non-immune pathways, such as autophagy, in response to knockdown or up-regulation of *Rel*, may contribute to the toxicity observed in *Gba1b*^-/-^ flies, for example by potentiating the autophagy impairment observed in GCase deficiency.

Taken together, our results highlight that potential therapeutic strategies aimed at modulating the immune system in *GBA1*-associated disorders, such as GD and PD, need to be approached with caution. We hypothesise that innate immunity is necessary to respond to the increased exposure to an altered gut microbiome in *GBA1* deletion, but that as the fly ages and the immune activation becomes chronic, its effects become detrimental at the organism level. Further work is now required to unravel the temporal and tissue-specific nature of the immune responses in *GBA1* deficiency, to determine whether targeting the innate immune system at later stages in life and/or in a tissue specific manner might offer an effective approach to treat *GBA1*-associated diseases.

In conclusion, we demonstrate innate immune dysregulation, GI dysfunction, and microbiome dysbiosis in a fly model of GCase loss-of-function. We show that the intestinal microbiota stimulates local gut and systemic innate immune responses in *Gba1b*^*-/-*^ flies. We also reveal that removal of the microbiome, or improvement of autophagy, but not chronic elimination of IMD signaling, improves survival as well as gut and locomotor phenotypes. These insights have the potential to lead to the development of novel long-awaited therapeutic approaches in the treatment of *GBA1*-associated disorders.

## Material and methods

### *Drosophila* stocks and handling

All flies were backcrossed at least 6 generations into the *w*^1118^ background to create isogenic background lines. Flies lacking *Gba1b* (*Gba1b*^*-/-*^) and *Gba1a* (*Gba1a*^*-/-*^) were previously described [22]. The *Gba1b* CRIMIC Trojan-Gal4 (BL#78943), *Rel*^E20^ (BL#755714), UAS-*Rel* (BL#755778), Tub-GAL4 (BL#5138) and Cg-GAL4 (BL#7011) lines were obtained from the Bloomington Stock Centre (Indiana, USA). All *Drosophila* stocks and experiments were maintained at 25°C on a 12:12 hour light: dark cycle at 60% humidity on a standard sugar-yeast medium (SYA) (50 g/L sugar, 100 g/L autolysed yeast, 15 g/L agar) containing preservatives (3 g/L nipagin and 3 ml/L propanoic acid). *Gba1b* knockout flies were kept in mixed populations with control flies for several generations prior to setting up all experiments, except for those involving the infection with *Lactobacillus plantarum*. This was performed to reduce the confounding effects of environmental conditions.

All experiments were set up using ‘egg squirt protocols’ to ensure that all experimental flies were raised at similar larval densities (~300 eggs per bottle). Following eclosion, flies were transferred to fresh food for a 48-hour mating period. Under CO_2_ anaesthesia, flies were then divided into 15 female flies per vial.

### RNA sequencing analysis

Next generation sequencing was performed by the Glasgow Polyomics (UK) facility. Using our in-house protocol, RNA was isolated from the *Gba1b*^*-/-*^ heads and the heads of aged-matched *w*^1118^ controls at 1 week and 3 weeks of age. For each sample time point, five replicates of 30 flies were used. The twenty samples were PolyA-enriched and the libraries were sequenced on a MiSeq Illumina instrument as 75 bp paired-end reads generating a total of 25 million reads. The mean output per sample was 10 million reads. Reads were mapped to the *Drosophila melanogaster* genome (downloaded from FlyBase.org, version dmel_r6.31_FB2019_06) using STAR v2.7.3a [78] (https://doi.org/10.1093/bioinformatics/bts635) [default parameters], plus the ENCODE options for ‘—alignIntronMin’, ‘—alignIntronMax’ and ‘—outFilterMultimapNmax’. Following reads sorting by gene name using STAR, featureCounts v.2.0.0 [79] (https://doi.org/10.1093/bioinformatics/btt656) [default parameters] was used to extract gene counts as per the transcriptome annotations (downloaded from FlyBase.org, version dmel_r6.31_FB2019_06/gtf). Differential gene expression analysis was performed for each time point independently vs the aged-matched controls using DESeq2 [80] (https://doi.org/10.1186/s13059-014-0550-8) [default parameters]. The generated p values were adjusted for multiple testing using the procedure of Benjamini and Hochberg [81]. Gene lists were created by filtering for lowly expressed genes (5 reads across all five replicates) and using absolute cut-offs for *p-value* <0.05 and log2 fold change ≥2 or log2 fold change ≤2 for up-regulated and down-regulated genes, respectively. Common genes at both time points were obtained by merging together the significant gene lists. These were further filtered by log2 fold change ≥2 at the latter time point generating common genes. Genes were visualised as a heat map created using the ‘heatmply’ R package v.1.1.0. Gene ontology (GO) enrichment analysis was performed using g:Profiler [82] (https://doi.org/10.1093/nar/gkz369).

### Quantitative RT-PCR

Total RNA was extracted from 6 whole flies, 25 heads, 10 dissected midguts or 10 headless bodies of adult flies at each time point using Trizol (Invitrogen) according to the manufacturer’s instructions. 4 μg of total RNA for each sample was subjected to DNA digestion using DNAse I (Invitrogen), immediately followed by reverse transcription using the Superscript II system (Invitrogen) with oligo(dT) primers. Quantitative RT-PCR was performed using the QuantStudio 6 Flex detection system (Applied Biosystems) and SYBR Green (Invitrogen) as per the manufacturer’s instructions. Each sample was analysed in duplicate, and the values are the mean of at least 4 independent biological repeats. The primers used were as follows:

**Table:** 

**Primer**	**Sequence (5’–3’)**
*Tub84B*_F	TGGGCCCGTCTGGACCACAA
*Tub84B*_R	TCGCCGTCACCGGAGTCCAT
*DptA*_F	GCTGCGCAATCGCTTCTACT
*DptA*_R	TGGTGGAGTGGGCTTCATG
*Drs*_F	GTACTTGTTCGCCCTCTTCG
*Drs*_R	TTAGCATCCTTCGCACCAG
*TotA*_F	TTCCGACGAAGATCGTGAGG
*TotA*_R	CTGGGTGCTATTGATTTTGGAGT
*Att_* F	GACACAATCTGGATGCCAAG
*AttA_*R	AATCCAGACCAGCTCCATTC
*PGRP-Sc2*_F	TGGCAAACAAAGCTCTCATC
*PGRP-Sc2*_R	ACGGCGTAGCTCAGGTAGTT
*CecC_*F	GGTTGGCTGAAGAAACTTGG
*CecC_*R	TTCCCAGTCCTTGAATGGTT
*Dro*_F	TCGAGGATCACCTGACTCAA
*Dro_*R	ATGACTTCTCCGCGGTATG
*Draper_*F	TGTGATCATGGTTACGGAGGAC
*Draper_*R	CAGCCGGGTGGGCAA
27_F	GAGAGTTTGATCCTGGCTCAG
1495_R	CTACGGCTACCTTGTTACGA

### Longevity and climbing assays

Flies were transferred to vials containing fresh food three times a week throughout life. The number of dead flies found during each transfer was recorded. Lifespan curves were analysed using a log-rank test. For climbing assays, 75 flies of each genotype were housed in groups of 15 in plastic vials held in a Drosoflipper (http://drosoflipper.com/). While video recording, the flies were tapped to the bottom and allowed to climb for 30 seconds. The numbers of flies in the top, middle and bottom thirds of each vial at 30 seconds were scored. A performance index was calculated as previously described [83].

### Germ-free (GF) conditions

Flies were rendered GF as previously described [84]. Axenic food was prepared with 50 mg/L tetracycline (Sigma-Aldrich, T3258) and 400 mg/L streptomycin (Sigma-Aldrich, S9137). In brief, embryos were collected on grape juice agar plates for 24 hours and transferred to a nylon basket. Embryos were dechorionated in 50% bleach for 2 minutes, washed twice in 70% EtOH for 1 minute and then washed in dH_2_O for 10 minutes. Dechorionated embryos were then transferred onto antibiotic food. Flies were processed at 3 weeks of age.

### Western blot analysis

For Western blotting, 10 fly heads or 8 guts per sample were homogenized in 2× Laemmli loading buffer (100 mM Tris 6.8, 20% glycerol, 4% SDS) containing 5% β-mercaptoethanol and then boiled for 5 minutes. Approximately 8 μl of protein extract was loaded per lane. Proteins were separated on precast 4%–12% NuPage Bis-Tris gels (Invitrogen) and transferred to a PVDF membrane. The membranes were then blocked in 5% BSA in TBST (TBS with 0.05% Tween 20) for 1 hour at room temperature, after which they were probed with primary antibodies diluted in 5% BSA in TBST overnight at 4°C. Blots were developed using the ECL detection system. Primary antibodies used: mouse DHSB Draper 5D14 (1:500); rabbit anti-GABARAP (ab109364, 1:2000); rabbit anti-Ref(2)P (ab178440, 1:500). The secondary antibody (Abcam) was diluted 1:10.000 (goat anti-mouse: ab6789 or goat anti-rabbit: ab672) in 5% BSA in TBST for 1 hour at room temperature. Bands were visualized with Luminata Forte (Millipore). All blots were imaged with ImageQuant LAS4000 (GE Healthcare Life Science). Quantification was performed using the ImageJ program (National Institutes of Health).

### Immunofluorescence microscopy

Tissue dissections were modified from [85]. Brains, guts or fat bodies were dissected in PBS and fixed, nutating in 4% paraformaldehyde (in PBS) for 20 minutes. Dissected tissues were washed twice with PBS-T (PBS, 0.5% TritonX), followed by 3x 20-minutes washes in PBS-T. Tissues were then incubated in block solution (PBS-T with 5% H1 horse serum (Gibco) for 1 hour before transferral to block containing primary antibody overnight at 4°C. Tissues were then washed twice in PBS-T followed by 3x 20 minutes in PBS-T and transferred to block solution containing secondary antibody overnight at 4°C. Tissues were then washed twice in PBS-T, followed by 3x 20 minutes in PBS-T and mounted in Vectashield antifade mounting medium with DAPI (Vector Labs, H1200). For experiments co-staining alongside mCherry, antibody incubations were reduced to 2 hours at room temperature to preserve mCherry signal. Antibody working concentrations were as follows: rabbit anti-Phospho-Histone H3 (Cell Signalling 9701, 1:100), mouse anti-Draper 5D14 (DSHB, 1:500), mouse anti-Repo 8D12 (DSHB, 1:200), mouse anti-Elav 958A9 (DSHB, 1:200), rabbit anti-Ref(2)P (ab178440, 1:200), mouse anti-FK2 (Sigma 04263, 1: 800). The secondary antibodies used: goat anti-mouse Alexa488 (A11001) and goat-rabbit Alexa568 (A11036), used at 1:250.

### LysoTracker staining

Fly guts were dissected in PBS and stained with LysoTracker, an acidophilic dye that labels lysosomes (LysoTracker Red DND-99; Thermo Fisher Scientific; 1:2000) and Hoeschst 33342 (Sigma, 1mg/ml; 1:1000) for 3 minutes. Immediately after staining, guts were washed 2x for 5 min with PBS, mounted in Vectashield and imaged.

### DptA-LacZ staining

DptA-LacZ expressing abdomens were dissected in ice-cold PBS and immediately fixed in 4% PFA for 20 minutes. Abdomens were washed in PBS and transferred into staining solution (10 mM potassium ferricyanide, 10mM potassium ferrocyanide, 1mM MgCl2, 150mM NaCl, 0.1% Triton in PBS), pre-warmed to 37°C and containing 1:20 X-gal (1 mg/ml in DMSO) and incubated overnight in the dark. Abdomens were washed 3x in pre-warmed PBS, mounted and immediately imaged on a Leica M165C light microscope. LacZ intensity was quantified by splitting RGB channels, inverting the image and quantifying the entire abdomen, with the background subtracted.

### Gut transit time and faecal deposit characterisation

Flies were placed on a SY diet containing 0.5% Bromophenol Blue (BPB) (Sigma-Aldrich, B5525- 10G) for 36 hours, then transferred into empty glass vials every 2 hours. Faecal deposits were then manually counted at each timepoint and assessed for ROD morphology.

### Gut permeability analysis (Smurf assay)

Gut barrier efficiency was analysed by placing flies on blue food prepared using 2.5% (w/v) FD&C blue dye n°1 (Fastcolors) as previously described [32]. Aged flies were kept on blue food for 6 days and subsequently scored for dye perfusion throughout the interstitial space.

### Food intake (Cafe assay)

In the capillary feeder (CAFE) assay, one single female fly was presented with liquid food using a 10 μL calibrated capillary per chamber (n = 12 per condition). Changes in liquid meniscus height were measured over three consecutive days. The feeding volume was calculated after background subtraction of measurements from empty chambers without flies.

### Image analysis

All images were acquired on a Zeiss LSM 700 confocal microscope and all settings were kept the same within an experiment. For Draper staining, a maximum intensity 10 μm z-stack with 1μm intervals encompassing the antennal lobes was taken. Regions of interest, encompassing the soma, were generated using the DAPI channel as a guide. Intensity relative to the antennal lobe neuropil was measured.

### Preparation of template DNA for 16S ribosomal RNA sequencing on fly midgut tissue

Extracted midguts were added to an Eppendorf tube containing 180 μL Lysis buffer (20 mM Tris-HCl pH 8.0, 2 mM EDTA pH 8.0, 1.2% Triton X-100 (Sigma-Aldrich), 20mg/mL fresh lysozyme from chicken egg (Sigma-Aldrich L7651)). 200 μL QIAGEN buffer AL was added to each Eppendorf tube containing 40 midguts. Following lysis with a Kontes pellet pestle, 20 μL proteinase K (QIAGEN) was added. The samples were then incubated at 56°C for 3 h. To remove any RNA, 10 μL of 10 μg/mL RNase A (Sigma-Aldrich R4875) was added and the samples were incubated at 37°C for 30 minutes. 200 μL ethanol was then added and the standard QIAGEN spin column protocol was followed. The DNA concentration was then assessed using a Nanodrop 2000 Spectrophotometer (Thermo Fisher Scientific) and sent to LC Sciences (Houston, USA) on dry ice for 16S/18S/ITS1/ITS2 RNA-sequencing.

### 16S ribosomal RNA sequencing

The V3-V4 region of the prokaryotic (including bacterial and archaeal) small-subunit (16S) rRNA gene was amplified with slightly modified versions of primers 338F (5’- ACTCCTACGGGAGGCAGCAG-3’) and 806R (5’- GGACTACHVGGGTWTCTAAT-3’). The 5’ ends of the primers were tagged with specific barcodes and sequencing universal primers. PCR amplification was performed in 25 μL of reactions containing 25 ng of template DNA, 12.5 μL of PCR premix, 2.5 μL of each primer, and PCR-grade water. The PCR conditions for amplifying the prokaryotic 16S fragments comprise the following steps: initial denaturation at 98°C for 30 seconds; 35 cycles of denaturation at 98°C for 10 seconds, annealing at 54°C/52°C for 30 seconds, and extension at 72°C for 45 seconds; and final extension at 72°C for 10 minutes. PCR products were confirmed with electrophoresis in 2% agarose gel. Ultrapure water was used as the negative control to exclude false positives. PCR products were purified by AMPure XT beads (Beckman Coulter Genomics, Danvers, MA, USA) and quantified by Qubit (Invitrogen, USA). The size and quantity of the amplicon library were assessed with Agilent 2100 Bioanalyzer (Agilent, USA) and Library Quantification Kit for Illumina (Kapa Biosciences, Woburn, MA, USA), respectively. PhiX control library (v3) (Illumina) was combined with the amplicon library (at a fraction of 30%). The libraries were sequenced on Illumina MiSeq (300 bṕ 2, pair-ended) using the standard Illumina sequencing primers.

### 16S ribosomal RNA sequencing data analysis

Paired-end reads were assigned to samples based on their unique barcodes before barcode and primer sequences were trimmed. The trimmed reads were merged using FLASH. Quality filtering on the raw tags were performed to obtain high-quality clean tags with *fqtrim* (v0.94). Chimeric sequences were filtered using *Vsearch* (v2.3.4). Sequences with ≥97% similarity were assigned to the same operational taxonomic units (OTUs) by *Vsearch* (v2.3.4). Representative sequences were chosen for each OUT, followed by taxonomic assignment using the RDP (Ribosomal Database Project) classifier. The differences of the dominant species in different groups and multiple sequence alignment were conducted by *mafft* software (v7.310). OTU abundance information was estimated after rarefaction with the least sequence number obtained for the project. Alpha diversity was applied for analyzing complexity of species diversity with 5 measurements, including Chao1, Observed species, Goods_coverage, Shannon and Simpson, which were calculated by QIIME (v1.8.0). Beta diversity was calculated by PCoA analysis to evaluate differences of samples in species complexity. Cluster analysis was performed by QIIME software (v1.8.0).

### 16S qPCR quantification of microbiota load

DNA was extracted from samples containing 5 female flies. Each fly was first sterilized with 70% ethanol to remove external bacteria and then washed with 1x PBS. After this step, the same protocol as described above was used to extract the DNA for the 16S Ribosomal RNA gene sequencing. qPCR was then performed on total genomic DNA to determine the bacterial load to fly DNA in each sample by normalizing it to the *Drosophila* GAPDH gene.

### CFUs and analysis of culturable bacteria

Culture-dependent methods were used to quantify and identify culturable bacteria present in the fly gut. Guts were dissected from female flies in Tris-HCl 50 mM, pH 7.5 and homogenized with a Kontes pellet pestle in 300μL de Man, Rogosa and Sharpe (MRS) broth. Each sample was then serially diluted and 50 μL from each dilution was plated onto MRS agar plates. Plates were incubated at 28°C for three days and isolated colonies present in each plate were counted using a digital colony counter (Fisher Scientific). Five colonies for each morphological type were re-streaked onto new plates. To identify each isolate, a PCR was performed using part of a colony as a DNA template and 16S universal primers (27F and 1495R). The amplified amplicon was cleaned up using a PCR purification kit (Qiagen) and sent to Source Biosciences for sequencing. Identified isolates were grown in liquid medium containing 25% of glycerol (v/v) and frozen at -80°C.

### Oral infection with Lactobacillus plantarum

Dissected guts from 6 *w*^1118^ control female flies (previously washed in EtOH and PBS) were homogenised and plated onto MRS agar medium and incubated at 25°C for two days. Colonies were then isolated and identified by 16S Ribosome Gene Sequencing (Source Bioscience). A single colony of *Lactobacillus plantarum* was then grown overnight in 400 ml of MRS broth at 25°C. The cell culture was centrifuged at 5000 rpm (10 min), washed in PBS and resuspended in 5 ml of 2.5% of sucrose. 200 μl of this suspension or 2.5% sucrose vehicle was pipetted onto 2 filter papers (Whatman filter paper Chroma circles 2.1 cm) in a vial containing 1.5% agarose. 3-week-old flies were transferred to vials containing either 2.5% sucrose and *Lactobacillus* or 2.5% sucrose alone. After 72 hours of feeding, fly death was scored.

### Micro-injection of flies with heat-killed bacteria

Newly eclosed flies were reared at 25°C on SY food until approximately 3 weeks of age. They were then subjected to an injection into the thorax with 32 nl heat-killed *Staphylococcus aureus* NCTC8325-4 (BAC) or PBS using a microinjector (Nanoject II; from Drummond Scientific).

### Statistical analyses

Statistical analyses were performed with Prism6 (GraphPad Software, USA). Data were tested for normal distribution and equal variance and accordingly analysed using adequate statistical tests as described in the legend of each figure. Statistical differences were considered significant at p<0.05. Log-rank test on lifespan data were performed in Microsoft Excel (template 351 available at http://piperlab.org/resources/) and data was plotted using Prism 6.

## Supporting information

S1 Fig Innate immune system genes are up-regulated in the heads of *Gba1b*^*-/-*^ flies compared to controls. **(A-B)** Volcano plots showing differentially expressed genes in the heads of *Gba1b*^*-/-*^ flies at 1 week **(A)** and 3 weeks **(B)** of age relative to their controls. Differential expression analysis was performed using DESeq2 (https://bioconductor.org/packages/release/bioc/html/DESeq2.html) and identified 11,324 differentially expressed genes at week 1 and 11,883 differentially expressed genes at week 3. Of these genes, 247 and 2545 were significant (S), (p-adjusted <0.05) at 1 week and 3 weeks, respectively. Of the significant genes (light grey) at 1 week, 34 were up-regulated (red) and 18 were down-regulated (blue). The significant genes at 3 weeks comprised of 178 up-regulated and 72 down-regulated genes. The up- and down-regulated genes were selected using the criteria p-adjusted <0.05 and log2 fold change of ± 2 (dotted lines). In addition to the non-significant (NS) genes (dark grey), the two fly *Gba1* genes, *Gba1a* and *Gba1b* are also highlighted and are up-regulated and down-regulated respectively at both time points. **(C-D)** The top 10 up-regulated (up) and down-regulated (down) genes ranked by the log2 fold change (Log2FC) at 1 week (**C**) and 3 weeks (**D**). **(E-F)** The top 50 KEGG pathways of the significant genes at both time points. KEGG pathways of the significant genes at week 1 (247, red) and at week 3 (2545, blue) were obtained using the KEGG database (https://www.genome.jp/kegg/pathway.html). Top pathways were calculated using the phyper package to compute significance (shown as p-value) between the number of genes in the query and the total genes associated with a particular pathway. Ranking was calculated as -log10 of the adjusted p-value and only the pathways with adjusted p-values <0.05 are shown.
(EPS)

S2 Fig Innate immune pathways are up-regulated peripherally in *Gba1b*^*-/-*^ flies. **(A-B)** Quantitative RT-PCR confirms up-regulation of the IMD (*DptA)* and JAK-STAT (*TotA*) reporter genes in the headless bodies of 3-week-old *Gba1b*^*-/-*^ flies compared to controls (*Drs*, p = 0.64; *DptA*, *p = 0.035; *TotA*, *p = 0.0102). All target gene expression levels are normalized to tubulin. Unpaired t-tests; data are presented as mean ± SD (n = 5–7 per genotype).
(EPS)

S3 Fig The innate immune pathways are not altered in the heads, guts or headless bodies of *Gba1a*^*-/-*^ flies. Quantitative RT-PCR analysis confirms that there is no significant up-regulation of the Toll (*Drs*), IMD (*DptA)* and JAK-STAT (*TotA*) reporter genes in **(A)** the heads **(B)** guts and **(C)** headless bodies of 3-week-old *Gba1a*^*-/-*^ flies compared to controls. All target gene expression levels are normalized to tubulin. Unpaired t-tests (normal distributed data) or Mann -Whitney test (non-normal distributed data); data are presented as mean ± SD (n = 5–6 per genotype).
(EPS)

S4 Fig *Gba1b* is expressed in the brain, fat body and gut. **(A)** The *Gba1b* gene expression pattern, as assessed by the expression of mCherry under the *Gba1b* endogenous promoter (red channel), overlaps with immunostaining for the glial marker Repo (green channel, top panel), but not with the neuronal marker Elav (green channel, bottom panel). Scale bars 50 μm. **(B)** Representative confocal image of *Gba1b* expression in abdominal tissues (white lines separate fat body from nephrocyte cells centrally). *Gba1b* expression, as indicated by mCherry expression driven under the *Gba1b* endogenous promoter (red channel), is highly expressed in fat body cells (magenta arrows) but not in nephrocytes (blue arrows). **(C)** *Gba1b* is highly expressed in the gut of 3-week-old flies (red channel).
(EPS)

S5 Fig *Gba1b*^-/-^ flies exhibit microbiome dysbiosis. **(A)** Heatmap showing the relative abundance of bacterial genera present in the midguts of *Gba1b*^-/-^ flies compared to their respective controls. Relative abundance is shown as a log2 transformed Z-scale, n = 5 per genotype. **(B)** No significant differences are found in food consumption between aged *Gba1b*^*-/-*^ and age-matched control flies using the CAFE assay. Unpaired t-test p = 0.7738, n = 12 per condition. **(C)** CFUs assay performed on gut tissue to determine the number of *Lactobacillus plantarum* bacteria fed to 3-week-old GF control and *Gba1b*^*-/-*^ flies. Both fly genotypes were fed with similar levels of bacteria (p = 0.3702, t-test). **(D)** Whole body *Drs* levels are significantly elevated in *Gba1b*^-/-^ flies compared to controls following an intrathoracic injection of heat-killed *Staphylococcus aureus* (*Gba1b*^*-/-*^ 0 vs 24 hrs **p = 0.00424; 24 vs 96 hrs ***p = <0.0001). Fold change in *Drs* expression between PBS and bacterial injection is shown normalized to tubulin. Two-way ANOVA followed by multiple comparison tests; data are presented as mean ± 95% confidence intervals (n = 4–6 per condition). **(E)** Lifespan of *Gba1b*^*-/-*^ and control flies after an intrathoracic injection of heat killed *Staphylococcus aureus* or PBS vehicle at 3 weeks of age. The survival curves demonstrate that there is no significant difference in lifespan of control flies treated with either bacterial or PBS injection (p = 0.062). *Gba1b*^*-/-*^ flies display reduced survival following heat-killed bacterial injection compared to flies receiving PBS vehicle injection (p = 2.5x10^-11^). Log-rank tests, n = 108–140 flies per condition.
(EPS)

S6 Fig Germ-free treatment decreases innate immune signalling without altering the autophagy defects in *Gba1b^-/-^* flies. **(A)** *Gba1b*^-/-^ fly heads display significant reduction of *AttA* (**p = 0.0045), *PGRPSc2* (***p<0.0001), *CecC* (*p = 0.0367) and *Dro* (*p = 0.0181) under GF conditions. Two-way ANOVA followed by uncorrected Fisher’s multiple comparison tests. **(B)** Western blot analysis on the fly heads of 3-week-old *Gba1b*^-/-^ and control flies raised under GF and non-GF conditions shows no significant difference in Ref(2)P or the ratio of Atg8a-II/Atg8a-I between the two conditions for both genotypes. Two-way ANOVA followed by uncorrected Fisher’s multiple comparison tests; data presented as mean ± SD. **(C)** LysoTracker staining and immunostaining for Ref(2)P and Ubiquitin (Ub) in brains of control and *Gba1b*^-/-^ flies. Under non-GF conditions there is an increase in LysoTracker positive puncta (***p<0.0001) and percentage of puncta area (***p<0.0001) in the brains of *Gba1b*^-/-^ flies compared to control fly brains. Ref(2)P and Ub puncta are also increased in the brains of *Gba1b*^-/-^ flies (***p<0.0001) compared to control fly brains. No alterations in these parameters are observed under GF conditions for both genotypes. Two-way ANOVA followed by Tukey’s multiple comparison tests; data are presented as mean ± SEM. Scale bar 100μM.
(EPS)

S7 Fig Modulation of the IMD pathway is toxic to *Gba1b*^-/-^ flies. **(A)** *Gba1b*^-/-^, *Rel*^E20^ double mutant flies display reduced levels of *Drs* compared with *Gba1b*^-/-^ flies (***p = 0.0005). There are also significant differences for the following comparison: Control vs *Gba1b*^-/-^ **p = 0.0015; *Gba1b*^-/-^ vs *Rel*^E20^ *p = 0.049. One-way ANOVA followed by Tukey’s multiple comparison test, data are presented as mean ± SD (n = 4 per condition). **(B)** Oral infection with *Lactobacillus plantarum* leads to a high mortality rate of *Gba1b*^-/-^, *Rel*^E20^ flies. It also moderately affects *Gba1b*^-/-^ and Rel^E20^ single mutants but not control flies. **(C)** Overexpression of Rel in the fat body using the CG-Gal4 driver results in higher expression levels of the *Rel* transcript in the headless bodies of CG>*Rel* (*p = 0.014) and CG>*Rel*, *Gba1b*^-/-^ flies (**p = 0.0013) compared to the driver alone in control and *Gba1b*^-/-^ backgrounds. One-way ANOVA followed by multiple comparison tests, data are presented as mean ± SD (n = 4,5 per condition). **(D)** qRT-PCR analysis reveals increased levels of *TotA* expression in CG>*Rel*, *Gba1b*^-/-^ flies (***p<0.0001) versus the driver alone in the *Gba1b*^-/-^ background. There are also significant differences in the expression of *TotA* in Cg, *Gba1b*^-/-^ flies versus CG driver alone (*p = 0.0153). One-way ANOVA followed by multiple comparison tests, data are presented as mean ± SD (n = 4,5 per condition).
(EPS)

S8 Fig Rapamycin stimulates autophagy in control and *Gba1b*^*-/-*^ fly guts. **(A)** Immunobloting for Atg8a in the fly gut in 3-week-old flies treated with Rapa shows a significantly increased ratio of Atg8a-II/Atg8a-I upon drug treatment in *Gba1b*^*-/-*^ flies but not in treated controls (***p = 0.001). Two-way ANOVA followed by Fisher’s multiple comparison tests. **(B)** The number of LysoTracker positive puncta increases in the gut of 3-week-old mutant and control flies treated with Rapa.
(EPS)
